# Sex-specific bone architectural deficits among older adults living with HIV: a cross-sectional study from Zimbabwe

**DOI:** 10.1093/jbmr/zjag082

**Published:** 2026-05-12

**Authors:** Mícheál Ó Breasail, Kate A Ward, Tadios Manyanga, Hannah Wilson, Camille Pearse, Anya Burton, Lucy Gates, Chris Grundy, Anthony Muchai Manyara, Denekew Tenaw Anley, Cynthia Kahari, Tafadzwa Madanhire, Peter R Ebeling, Rashida A Ferrand, Celia L Gregson

**Affiliations:** Department of Medicine, School of Clinical Sciences, Faculty of Medicine, Monash Medical Centre, Nursing and Health Sciences, Monash University, Clayton, VIC, Australia; Global Musculoskeletal Research Group, Musculoskeletal Research Unit, Bristol Medical School, University of Bristol; MRC Lifecourse Epidemiology Centre, Human Development and Health, University of Southampton, Southampton, UK; MRC Unit, The Gambia @ London School of Hygiene and Tropical Medicine, Banjul, The Gambia; The Health Research Unit Zimbabwe, Biomedical Research and Training Institute, Harare, Zimbabwe; Global Musculoskeletal Research Group, Musculoskeletal Research Unit, Bristol Medical School, University of Bristol; MRC Lifecourse Epidemiology Centre, Human Development and Health, University of Southampton, Southampton, UK; Global Musculoskeletal Research Group, Musculoskeletal Research Unit, Bristol Medical School, University of Bristol; MRC Lifecourse Epidemiology Centre, Human Development and Health, University of Southampton, Southampton, UK; MRC International Statistics and Epidemiology Group, London School of Hygiene and Tropical Medicine, London, UK; Global Musculoskeletal Research Group, Musculoskeletal Research Unit, Bristol Medical School, University of Bristol; Department of Medicine, School of Clinical Sciences, Faculty of Medicine, Monash Medical Centre, Nursing and Health Sciences, Monash University, Clayton, VIC, Australia; Department of Oncology, Medical Physics and Imaging Sciences, Faculty of Medicine and Health Sciences, University of Zimbabwe, Harare, Zimbabwe; Global Musculoskeletal Research Group, Musculoskeletal Research Unit, Bristol Medical School, University of Bristol; Department of Medicine, School of Clinical Sciences, Faculty of Medicine, Monash Medical Centre, Nursing and Health Sciences, Monash University, Clayton, VIC, Australia; The Health Research Unit Zimbabwe, Biomedical Research and Training Institute, Harare, Zimbabwe; Clinical Research Department, Faculty of Infectious and Tropical Diseases, London School of Hygiene and Tropical Medicine, London, UK; Global Musculoskeletal Research Group, Musculoskeletal Research Unit, Bristol Medical School, University of Bristol; The Health Research Unit Zimbabwe, Biomedical Research and Training Institute, Harare, Zimbabwe

**Keywords:** ANALYSIS/QUANTITATION OF BONE, Bone QCT/microCT, osteoporosis, general population studies, AGING

## Abstract

People with HIV (PLWH) are living longer with antiretroviral therapy (ART) but HIV increases the risk of age-associated diseases including osteoporosis. A cross-sectional study of men and women ≥40 years was conducted in Harare, Zimbabwe. Sociodemographic data, HIV status/treatment, and anthropometry were collected. Outcomes were peripheral quantitative computed tomography (pQCT): total volumetric bone mineral density v(BMD), trabecular vBMD, cross-sectional area (CSA), compressive bone strength (BSIc), cortical vBMD, cortical thickness, proximal CSA, and polar stress-strain index (pSSI). Sex-stratified linear regression determined differences by HIV status, minimally adjusted for age and wealth-index (WI), and further adjusted for height and weight. Linear regression adjusted for age and WI assessed associations between HIV- and ART-duration, TDF-use, and viral suppression on bone. 1101 participants (PLWH 20.3%; Male 48.6%), aged mean(SD) 62.4(14.1) years, had pQCT data. HIV-related bone impairments, robust to full adjustment for age, wealth-index, height, and weight were observed. Men with HIV (MLWH) had lower radial total vBMD (-7.1% [-10.9; -3.2]%), trabecular vBMD (-11.3% [-16.9%; -5.6%]), and BSIc (-12.0% [-18.7%; -5.4]%) than HIV-negative men. Trabecular findings were similar in women with HIV (WLWH), plus lower cortical vBMD (-1.4 [-2.3; -0.6]%) and thickness (-7.2 [-11.1; -3.3]% were also seen. Tibial pQCT findings were broadly consistent. In WLWH , years since HIV-diagnosis were associated with lower radius total vBMD (-1.9% per-year [-3.2%; -0.6%]), trabecular vBMD (-4.2% [-6.5%; -2.0%]), BSIc (-3.9% [-6.2%; -1.6%]), cortical vBMD (-0.3% [-0.6%; -0.1%]), and thickness (-1.6% [-2.7%; -0.5%]). In MLWH, years since HIV-diagnosis were associated with smaller proximal tibia CSA (-1.0% [-1.9%; -0.2%]) and TDF-use with lower tibia cortical vBMD (-1.4% [-2.8%; -0.1%]). Older Zimbabwean PLWH have markedly lower trabecular vBMD compared to HIV-negative peers additional cortical compartment impairments were evident in women. These data add to the growing evidence that women with HIV in particular may be at increased risk of age-associated osteoporotic fragility fracture relatively early in their life-course.

## Introduction

East and Southern Africa, are home to the greatest number of people living with HIV (PLWH) globally.^1^ Today PLWH across the region can expect to live to midlife and beyond; widespread availability of antiretroviral therapy (ART) has transformed HIV into a chronic disease of ageing, characterized by age-associated non-infectious complications, such as osteoporosis among a high prevalence of other non-communicable diseases (NCDs).^2–4^ HIV affects bone largely via T and B cells depletion; damage to the latter reduces osteoprotegerin (OPG) production and increases the production of receptor activator of NF-κB ligand (RANKL).^5^^,^^6^ This altered OPG to RANKL ratio underpins HIV-related bone loss by upregulating bone resorption.^7^ In turn, ART initiation leads to T-cell repopulation and secretion of osteoclastogenic cytokines (including RANKL and tumor necrosis factor (TNF)). This is believed to contribute to an inflammatory state promoting osteoclastic bone loss common across different ART regimens.^7^ Together with these disease- and drug-specific challenges to bone, PLWH may also be impacted by social and behavioral factors associated with poorer bone health and increased fracture risk.^8^ Though notably these exposures vary between high- and low-income settings, e.g., smoking is uncommon in African women. Recent meta-analyses largely based on studies from high income settings have consistently found that PLWH are at greater fracture-risk than peers.^9–15^ The importance of maintaining bone health in PLWH across the lifecourse and in both high income and more resource-limited settings has become increasing recognized as a means of reducing fracture risk.^16–20^ However, data from older PLWH in the regions with the greatest HIV burdens are largely lacking and represent a major knowledge gap.

Recently, effects of HIV on bone in midlife African women living with HIV (WLWH) have begun to be reported.^21–24^ These data from southern Africa suggest WLWH have pre-existing bone deficits prior to menopause,^21^^,^^23^ lose more bone across menopause,^24^ and have a higher prevalence of osteoporosis and major osteoporotic fractures (MOFs) compared to HIV negative women.^23^ Taken together this suggests that as WLWH advance from midlife into older age, they will be more likely to develop osteoporosis and be at increased fracture-risk. In contrast, comparatively little is known about bone health in older African men living with HIV (MLWH), though dual energy x-ray absorptiometry (DXA) has shown rural-dwelling MLWH in South Africa have lower areal bone mineral density (aBMD) than uninfected peers.^3^ Although DXA is the clinical standard for osteoporosis diagnosis, as a two-dimensional modality (2D) it is incapable of quantifying compartment-specific (e.g. trabecular vs. cortical) bone density. In contrast, peripheral Quantitative Computed Tomography (pQCT) provides estimates of both trabecular and cortical volumetric bone mineral density (vBMD) in addition to bone strength estimates in the appendicular skeleton. We have previously reported pQCT assessed HIV-related differences in both trabecular and cortical vBMD, bone architecture, and estimated strength in midlife women from both urban South Africa (Soweto) and Zimbabwe (Harare).^21^^,^^25^ In Zimbabwe we observed large differences in trabecular vBMD by HIV status, while in both the South African and Zimbabwean studies we also saw some evidence of potential cortical compartment impairment in post-menopausal WLWH. These studies did not establish whether midlife MLWH were also at risk of HIV-related impairments in BMD and bone architecture as they age.

The current cross-sectional study took place in Harare, Zimbabwe, where HIV prevalence ranges from 27.4% at 40-44 years to 14.5% at 60-64 years.^26^ It was devised as a pre-planned secondary analysis within a large multi-country mixed-methods research program of bone health in older Africans^27^ focused on men and women aged 40 years and older thus building on our previous work in the region^21–23^^,^^28^ by including a) men, and b) an older female population. Within this pQCT-focused study our primary aim was to quantify HIV-specific differences in trabecular and cortical vBMD in PLWH relative to their peers without HIV; secondary aims were to 1) quantify HIV-related differences in pQCT assessed bone geometry, distribution and strength indices, and 2) in PLWH investigate whether HIV-specific factors are associated with poorer pQCT outcomes, and finally to 3) describe pQCT parameters by prevalent major osteoporotic fracture status in the entire sample and by HIV status.

## Materials and methods

This is pre-specified secondary analysis within the Fractures-E3 (Fractures in sub-Saharan Africa: epidemiology, economic impact, and ethnography) study, conducted in three countries in Africa, including Zimbabwe.^27^ The current study utilized a cross-sectional population-based study conducted between February and November 2022 in Harare, which aimed to recruit 168 women and 168 men in each of three age strata (40–54, 55–69 and ≥70 years), generating a total of 504 women and 504 men. A sample of 168 women and 168 men provided 90% power to detect an odds ratio of 2.0 for an association between a risk factor with 13% prevalence (e.g. HIV) and an outcome seen in 9% (e.g. spine fracture).^27^ The sampling strategy has been described in detail in the protocol paper.^27^

### Data collection

Socio-demographic data, including educational attainment and lifestyle information, and medical history were captured by questionnaire. A wealth-index was generated based on a principal component analysis of household assets.^29^ Tobacco use (smoking, snuff, or chewing) was self-reported as ‘current’, former’ or ‘never’. Alcohol consumption was obtained by self-report as number of drinks/week and converted to units of alcohol/week. Excess consumption was defined as >14 units/week. Low physical activity was defined as previously reported,^30^ in brief, self-reported activity was assessed using the International Physical Activity Questionnaire-Short Form^31^ from which metabolic equivalent (METS) min/week were calculated and converted to kcal/week. Low physical activity was defined as: men <383kcal/week and women <270kcal/week.^32^ Prior fracture history was obtained by self-report of a crack, break or fracture, and from lateral spine DXA scans (major osteoporotic fractures [MOF] defined as fragility fractures of the hip, spine, wrist and/or humerus). Vertebral fractures were quantified radiologically from lateral DXA. Cumulative MOFs for each individual were calculated from self-reported fracture and expert-read lateral spine DXA scans. Women currently having regular periods were classified as premenopausal, those having irregular periods and/or a period within the previous 12 months as perimenopausal, and women who had had no bleeding for ≥12 months as postmenopausal; those who reported hysterectomy (n = 12) or who were uncertain thereof (n = 12) were excluded from this classification. Medication and multivitamin use were recorded, including current ART regimen, menopausal hormone therapy (MHT), and any medicines affecting bone health (e.g. bisphosphonates, glucocorticoids).

### HIV testing

Participants who had never had an HIV test or had previously tested negative were offered HIV testing. Those with an established diagnosis of HIV were not re-tested. Those newly diagnosed in the research clinic and those with an established diagnoses were considered to be living with HIV.^27^ Positive diagnosis was based on confirmation by two different rapid point-of-care tests (Alere Determine® HIV-1/2 from Abbott Laboratories and Chembio® from Chembio Diagnostics, New York, Illinois)^27^. HIV viral load was measured for all PLWH, viral suppression was defined as <50 copies/mL^33^. Duration since HIV diagnosis and ART initiation were based on clinical records and/or self-report.

### Anthropometry

Two nurses measured height (cm) and weight (kg) using a Seca 213 stadiometer and Seca 875 digital scales (Seca Precision for Health, Seca Mechanical Floor Scales Class III, Hamburg, Germany) respectively, with the mean of both measurements calculated. Body mass index (BMI) was calculated (kg/m^2^).

### Peripheral quantitative computed tomography (pQCT) scanning

**Figure 1:**
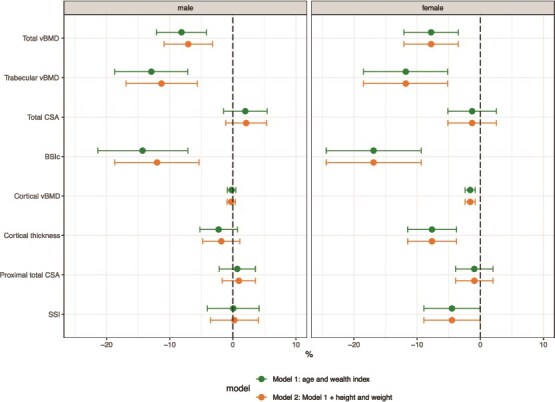
Estimates of differences in pQCT parameters at the radius by HIV status in a) men and B) women. Model 1 adjusted for age (yr) and wealth index; model 2 adjusted for age (yr), wealth index, height (m), and weight (kg). Tests for sex*HIV interactions were performed with no interactions p<0.05 detected. Abbreviations: vBMD, volumetric BMD; CSA, cross-sectional area; BSIc, bone strength index of compression; SSI, stress–strain index.

pQCT imaging was preformed using standard operating procedures as previously described in this setting.^21^ In brief, scans at the radius (at 4% and 33% of the limb length proximal to the distal endplate) and tibia (at 4%, and 38% of the limb length proximal to the distal endplate), were acquired on an XCT 2000L^TM^ (Stratec Medizintechnik, Pforzheim, Germany). Scans were obtained at a voxel size of 0.5 × 0.5 mm and 2 mm slice thickness with a CT scan speed of 30 mm/s and scout view scan speed 40 mm/s. Figure 1. provides an overview of the distal and proximal sites measured at the tibia. All pQCT images were processed using the manufacturer’s software (Stratec XCT version 6.2). At distal trabecular-rich sites, CALCBD analysis calculated total cross-sectional area (CSA) and total vBMD and trabecular vBMD. CALCBD contour mode 1 (i.e., the threshold algorithm) excluded pixels in the region of interest (ROI) below a threshold of 180 mg/cm^3^; peel mode 1 (i.e., concentric peel) peeled the outer 55% of total CSA leaving the inner 45% CSA classed as trabecular bone. Bone Strength Index of Compression (BSIc, g2/cm^4^) was subsequently derived as the product of the total vBMD (g/cm^3^) squared and total CSA (cm^2^). At proximal cortical-rich sites (33%/38%), the CORTBD algorithm defined cortical parameters by removing all voxels within the ROI with an attenuation coefficient threshold of 710 mg/cm^3^ (separation mode 1). Cortical thickness was calculated using a circular ring model.^38^ Proximal total CSA was defined with an edge detection threshold of 280 mg/cm^3^. Polar stress-strain Index (pSSI), an estimate of bone strength, was obtained at a threshold of 280 mg/cm^3^ using cortmode 1.

All scans were qualitatively graded by radiographers through visual inspection to assess their suitability for analysis: scan slices with excessive movement (e.g. >2 on a scale of 0 [no motion] to 3 [unusable due to excessive motion] as used in previous studies^21^^,^^34–36^) or other artefacts, and scout views with incorrect reference line placement were excluded.

### Dual-energy X-ray absorptiometry (DXA)

DXA scans were performed using an iDXA Pro (GE Lunar, Waltham, MA, USA; software version 18) to assess the lateral thoracic and lumbar spine for vertebral (spine) fractures.

### Imaging quality assessment (QA) and quality control (QC)

Scanner performance during the study was monitored using standard manufacturer QA (daily) and QC (weekly) protocols. Inter-operator precision has been previously reported in this setting based on repeat scans from 30 adults with the root mean squared co-efficient of variation (RMS-CV%) for pQCT ranging between 0.8-7.3% at the radius and 0.8-4.8% at the tibia.^21^ Specifically, in relation to our primary outcomes of trabecular and cortical vBMD these were: 3.4% and 0.8%, at the radius; 1.8% and 0.8% at the tibia.

### Ethical considerations

The study obtained ethical approvals from the Biomedical Research and Training Institute Institutional Review Board (Ref: AP152/2019), and the Harare Central Hospital Ethics Committee (Ref: HCHEC 181119/66), as well as the Medical Research Council of Zimbabwe (Ref: MRCZ/A/2551). Informed written consent was collected from all participants.

### Statistical analysis

All statistical analyses were performed using RStudio (2023.06.1 Build 524), R v4.3.1 (R Foundation for Statistical Computing, Vienna, Austria; https://www.r-project.org/). Participant characteristics are tabulated by sex and HIV status with continuous descriptive data, where normally distributed, summarized as mean(SD) or otherwise presented as median[IQR] if skewed and with categorical data expressed as counts and percentages (n[%]). Independent sample t-tests and Pearson’s Chi-squared tests were used for hypothesis testing for normally distributed continuous and categorical variables, respectively. Trabecular and cortical vBMD were considered primary outcomes, with the remaining pQCT measures of total vBMD, distribution (CSAs and cortical thickness) and strength (BSIc and pSSI) investigated as secondary outcomes. The linear relationship between pQCT parameters and age within sex and HIV strata was plotted. pQCT data were also tabulated as mean(SD) by sex and HIV status, with differences by HIV status investigated using independent sample t-tests.

Sex-stratified linear regression was used to determine relationships between HIV status and pQCT bone outcomes, controlling for *a-priori* confounders, age and wealth-index (Model 1), with then further adjustment for height and weight (Model 2). The use of alternative adjustments to account for body composition (i.e. BMI, fat mass and height, fat mass index) were explored but all models behaved similarly regardless of which confounder was included. Additional adjustments for lifestyle factors: low physical activity, glucocorticoid use, excess alcohol intake, and smoking history were also explored. In women, we also explored whether adjustment for menopause status in addition to age influenced findings. To allow us to compare the magnitude of between-group differences across different pQCT parameters, pQCT parameters (dependent variable) were natural log transformed, to normalize their distribution and allow the expression of differences by HIV status as symmetric percentages.^37^ We followed the approachs outlined by Cole et al 2000, where “the transform y = 100 log_e_x leads to differences, standard deviations and regression coefficients of y that are equivalent to symmetric percentage differences, standard deviations and regression coefficients of x”.^38^ Subsequently, sex*HIV interactions were explored in the fully adjusted model.

Sex-stratified linear regression determined the association between years since HIV diagnosis and pQCT measures, adjusted for age and wealth index (Model 1). Further adjustment for cumulative years on ART (Model 2) was performed to understand if this modified any relationship. Associations between current TDF use and viral suppression and pQCT measures were examined, with adjustment for age and wealth index. As above, in women, we further explored whether adjustment for menopause status, in addition to age, influenced findings.

Linear regression was used to determine mean (95%CI) differences in pQCT parameters by prior MOF status, without covariates (model 1) and adjusted for age, sex, and wealth index (model 2). These models were then stratified by HIV status. Symmetric percentage differences by MOF status were also computed, as above, to visualize differences in pQCT parameters on a comparative scale. Analyses limited to PLWH were performed adjusted for years since HIV diagnosis, and ART duration.

## Results

One-thousand one-hundred and one participants (M 48.6%), mean(SD) age 62.4 (14.1) years, had at least one usable pQCT scan (Figure S1, Table S1). Of 223 (20.3%) living with HIV, 15 were newly diagnosed by the research team. These ranged in age from 40 to 83 years, mean (SD) 55.5 (11.3) years. Of those with previously diagnosed HIV, 96.2% (200/208) were established on ART, and 89.7% (175/195) of those on ART had a viral load <50 copies/mL. On average, those with known HIV had been diagnosed at age 44.6 (10.4) years, had lived with HIV for 9.8 (5.0) years, and had been on ART for 9.4 (5.2) years. PLWH were, on average, a decade younger than those without HIV (54.2 (9.5) vs. 64.1 (14.3) years. PLWH of both sexes were taller though weight did not differ, though MLWH had lower BMI than men without HIV (Table 1). Women regardless of HIV status were predominantly postmenopausal (Table 1). No use of MHT or anti-osteoporosis medication was reported by any individual. Self-reported glucocorticoid use of ≥3 months was reported by 2.8% of participants (2.4% HIV-negative vs 4.5% PLWH). Multivitamin use was comparable by HIV status, approximately 8% of participants in both groups reported taking a supplement ≥3 times a week. Low physical activity was more common among HIV-negative participants (PLWH 42% vs HIV-ve 53%), though when stratified by sex this pattern was only evident in men (Table 1).

**Table 1:** Descriptive characteristics of Zimbabwean men and women aged 40 years and older. Data expressed as mean(SD) or count(%) unless stated otherwise.

Characteristic	Men without HIV (n = 428)	Men with HIV (n = 107)	*p*-value	Women without HIV (n = 450)	Women with HIV (n = 116)	*p*-value
Age, years	64.5 (14.9)	54.4 (9.3)	<0.001	64.5 (13.7)	54.0 (9.7)	<0.001
Age groups (n(%)) 40-54 years 55-69 years 70+ years	125 (29.2) 134 (31.3) 169 (39.5)	52 (48.6) 47 (43.9) 8 (7.5)	<0.001	121 (26.9) 136 (30.2) 193 (42.9)	63 (54.3) 43 (37.1) 10 (8.6)	<0.001
Education (n(%)) Primary or no education Secondary and above	126 (29.4) 302 (70.6)	19 (17.8) 88 (82.2)	0.021	217 (48.2) 233 (51.8)	32 (27.6) 84 (72.4)	<0.001
Socioeconomic tertiles (n(%)) T1 T2 T3	124 (29.0) 158 (36.9) 146 (34.1)	39 (36.4) 34 (31.8) 34 (31.8)	0.310	162 (36.0) 137 (30.4) 151 (33.6)	47 (40.5) 38 (32.8) 31 (26.7)	0.366
Alcohol use >14 units/week	55 (12.9)	16 (15.0)	0.672	6 (1.3)	4 (3.4)	0.247
Ever used tobacco	133 (31.1)	27 (25.2)	0.288	26 (5.8)	4 (3.4)	0.444
Low physical activity, n(%)	221 (53) ^(n = 415)^	41 (39) ^(n = 105)^	0.013	242 (55) ^(n = 443)^	53 (46) ^(n = 115)^	0.126
Height (m)	1.71 (0.07)	1.73 (0.06)	0.003	1.59 (0.07)	1.61 (0.06)	<0.001
Weight (kg)	68.0 (13.5)	66.0 (13.0)	0.150	73.3 (18.1)	72.7 (15.6)	0.699
BMI (kg/m^2^)	23.3 (4.2)	22.1 (4.0)	0.005	29.0 (6.5)	28.0 (6.2)	0.118
BMI categories (n(%)) Underweight (<18.5 kg/m^2^) Normal (18.5<25 kg/m^2^) Overweight (25<30 kg/m^2^) Obese (≥30 kg/m^2^)	43 (10.0) 252 (58.9) 103 (24.1) 30 (7.0)	18 (16.8) 68 (63.6) 18 (16.8) 3 (2.8)	0.042	(n = 447) 9 (2.0) 123 (27.3) 135 (30.0) 180 (37.9)	(n = 115) 4 (3.4) 32 (27.6) 44 (37.9) 35 (30.2)	0.170
Previous MOF, n (%)	41 (9.6)	8 (7.5)	0.626	50 (11.1)	11 (9.5)	0.737
Prevalent vertebral fracture, n(%)	34 (8.1)	5 (4.9)	0.373	40 (9.2)	6 (5.4)	0.260
Post-menopausal*	-	-	-	351 (82)	82 (74.1)	0.074
						
*HIV-specific characteristics*	n			n		
Age at HIV diagnosis (years)	107	45.9 (10.0)		115	45.2 (11.7)	
Years since HIV diagnosis, median[IQR]	107	9.0 [4.7; 12.4]		115	9.4 [5.2; 13.4]	
Taking ART (n (%))	107	97 (90.7)		116	103 (88.9)	
Years on ART (median[IQR])	106	8.0 [4.0; 12.0]		115	9.0 [4.0; 13.0]	
Current TDF (n (%))	107	89 (91.8)		116	94 (91.3)	
Viral load in copies/mL (n(%)) <50 ≥50	104	90 (86.5) 14 (13.5)		112	90 (80.4) 22 (19.6)	

p-values presented from unpaired t-tests for continuous variables unless otherwise stated. Pearson’s chi -square test used for categorical variables. ART = anti-retroviral therapy, BMI = body mass index, FMI = fat mass index, TDF = tenofovir

^*^12 women with known hysterectomy and 12 women with uncertain hysterectomy history not included. Low physical activity categorized as: men <383kcal/week and women <270kcal/week

### Unadjusted differences in pQCT bone measures by HIV status and age

At the distal radius, advancing age was associated with lower vBMD and BSIc in both sexes, except for Trabecular vBMD in MLWH (Figure S2A). At the proximal radius, age was associated with lower Cortical vBMD and Cortical thickness in all, with a trend towards lower pSSI in women (Figure S2B). In men without HIV and all women, age was associated with lower tibial total vBMD, trabecular vBMD and BSIc (Figure S3A). In all, age was associated with lower cortical vBMD, and in men without HIV and all women, lower cortical thickness and pSSI (Figure S3B). HIV by age interactions were observed only in distal and proximal tibia CSA in WLWH, in whom bone area was greater with each year of age (p = 0.03, and p = 0.009, respectively). Unadjusted differences in bone by HIV status were few, and largely in men only (Table S1).

### Adjusted differences in pQCT bone measures between those with and without HIV

In both sexes, controlling for age and wealth-index, distal radius and tibia vBMD (total and trabecular) and BSIc were lower in PLWH (Figure 1, Figure S4). These impairments were robust to further adjustment for height and weight at the load-bearing distal tibia and non-load-bearing distal radius (Figure 1, Figure S4). At the proximal radius and tibia no HIV-related bone impairments were found in MLWH (Figure 1, Figure S4). Whereas, WLWH had lower radial cortical vBMD, cortical thickness, and pSSI compared to HIV-negative peers, including after age and wealth-index adjustment (Figure 1). Further adjustment for weight and height had minimal attenuation, apart from pSSI where the association weakened (Figure 1). In women, tibial findings were similar to those at the radius, other than tibial pSSI which remained lower after adjustment (Figure S4).

Additional adjustments for glucocorticoid use, physical activity, excessive alcohol consumption, and smoking did not alter the associations seen (data not shown). In women, adjusting for menopause status in addition to age did not change the associations (data not shown).

### HIV-specific factors and bone architecture

**Figure 2:**
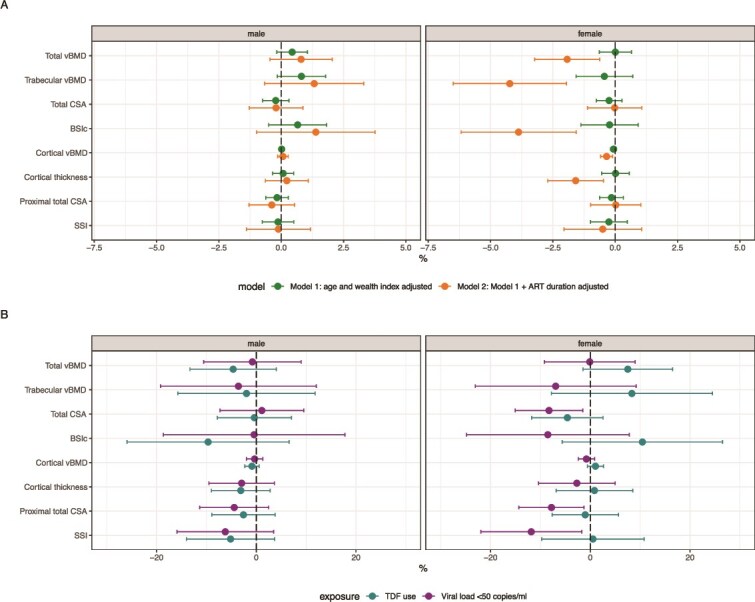
Associations between HIV-specific factors and radial pQCT parameters in Zimbabwean men and women living with HIV. Panel a) associations between time since diagnosis and pQCT parameters adjusted for age and wealth index (model 1) and additionally adjusted for years of ART use (model 2). Panel B) associations between TDF-use suppressed viral load (<50 copies/mL) with pQCT parameters adjusted for age and wealth index. vBMD = volumetric bone mineral density, CSA = cross-sectional area, BSIc = bone strength index of compression, SSI = stress-strain index.

Time since HIV diagnosis was not associated with pQCT measures in PLWH in age and wealth-index adjusted models (Figure 2A, Figure S5A). However, after adjustment for ART duration, MLWH who had lived longer since HIV diagnosis had smaller proximal tibial CSA (-1.0% [-1.9%; -0.2%] (Figure 5SA). Whilst, following adjustment for ART duration, in WLWH each year since HIV diagnosis was associated with lower radius total vBMD (-1.9% [-3.2%; -0.6%]), trabecular vBMD (-4.2% [-6.5%; -2.0%]), BSIc (-3.9% [-6.2%; -1.6%]), cortical vBMD (-0.3% [-0.6%; -0.1%]), and cortical thickness (-1.6% [-2.7%; -0.5%]) (Figure 2A). Further adjustment for ART duration did not change associations with tibial pQCT parameters in WLWH (Figure 5SA)

TDF-use was associated with lower tibial cortical vBMD (-1.4% [-2.8%; -0.1%]) in MLWH (Figure 5SB), but not in WLWH (Figure 2B, Figure 5SB). In WLWH, viral suppression was associated with lower distal (-8.3% [-15.0%; -1.5%]) and proximal (-7.8% [-14.3%; -1.3%]) radius CSA and radius pSSI (-11.8% [-22.0%; -1.7%]) (Figure 2B).

Further adjustment for menopause status in addition to age did not change the associations above (data not shown).

### Differences in pQCT measures by history of prior fracture

**Figure 3:**
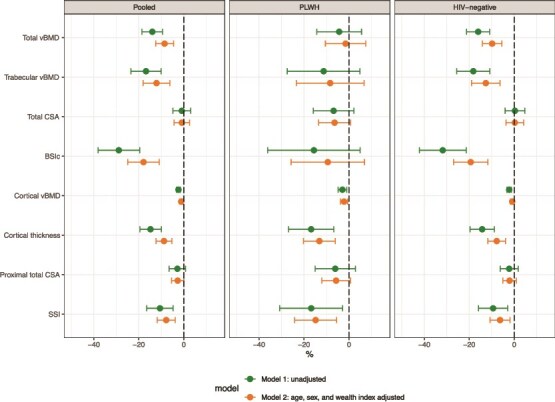
Difference in radial pQCT parameters in those with and without a prior major osteoporotic fracture (MOF) in Zimbabwean older adults. Data expressed as mean(95% CI) percent difference between those with a history of MOF and those without. Model 1, unadjusted; model 2 adjusted for age, sex, and wealth index. PLWH = people living with HIV vBMD = volumetric bone mineral density, CSA = cross-sectional area, BSIc = bone strength index of compression, SSI = stress-strain index.

Mean (95% CI) estimates of radial and tibial pQCT parameters, in addition to mean absolute between-group differences, by MOF status for the all participants, PLWH, and HIV-negative participants are tabulated in Supplementary Tables S2–S4 unadjusted (model 1) and adjusted for age, sex, and wealth index (Model 2). Participants with prevalent MOF had lower radius total vBMD (-14.0%[-18.6%; -9.4%]), trabecular vBMD (-16.8% [-23.5%; -10.1%]), BSIc (-28.9% [-38.1%; -19.6%]), Cortical vBMD (-2.4% [-3.3%; -1.5%]) and pSSI (-10.6% [-16.5%; -4.8%]) (Figure 3). These relationships within HIV strata followed a similar pattern though among PLWH there was substantially greater variation as evidenced by wider confidence intervals (Figure 3). Adjustment for age, sex, and wealth index had minimal attenuation on estimates for all participants or within HIV strata (Figure 3). Tibial data were mostly consistent with those at the radius (Figure S6). Sensitivity analysis limited to vertebral fracture only, showed consistent findings (data not presented).

In PLWH, further accounting for HIV-specific factors of i) time since diagnosis, and ii) time on ART in model 2, had little influence on the observed relationships between MOF status and pQCT parameters (Figure S7, Table S5).

## Discussion

This study demonstrates that older PLWH in Zimbabwe have sex-specific bone architectural impairments impacting bone density, distribution and strength. These were most pronounced in trabecular bone density, which was approximately 10% lower in PLWH of both sexes. Cortical compartment impairments were only apparent in women with HIV, where cortical density was 1.5% lower and cortices 7.5% thinner. Furthermore, in women, each year lived with HIV was associated with approximately 4% lower trabecular vBMD and 0.4% lower cortical vBMD at the radius, independent of age and ART duration. Given the radius is a common site of postmenopausal osteoporotic Colles’ fracture, this raises concerns about future osteoporotic fracture-risk in WLWH, as ageing women are already at higher fracture risk than men. Overall, pQCT measures of bone density and strength were consistently lower in those who had had a prior MOF; however, when stratifying by HIV status these relationships were weaker in PLWH suggesting other factors may contribute to fracture beyond bone architecture.

In high income countries, higher rates of osteoporosis and fracture are well-documented in PLWH,^2^^,^^12^^,^^13^ but there are few data regarding older PLWH in African countries, where the burden of HIV remains high and fracture risk factors may differ due to differences in sociodemographic and the underlying epidemiology of HIV in this region.^8^ This study highlights the extent of sex- and compartment-specific bone architecture deficits in Zimbabwean PWLH, despite being established on ART with good viral suppression. There are no comparable compartment-specific data in older African MLWH, though in neighboring South Africa older MLWH have been shown to have lower DXA-measured femoral neck aBMD than those without HIV^3^. Interestingly, the magnitude of lower trabecular vBMD in both sexes were similar to those recently reported in midlife Zimbabwean WLWH^21^. This may indicate that HIV influences more metabolically active trabecular bone in keeping with a number of DXA studies in midlife WLWH which observed greater differences in LS aBMD Z/T-scores compared to other skeletal sites.^23^^,^^39–41^ In older African men no HIV-related cortical compartment impairments were observed, whereas lower cortical vBMD and cortical thickness in older WLWH was evident and exceeded estimates previously described in the midlife population.^21^ Evidence of poorer compressive (BSIc) bone strength was found in both sexes driven by lower total vBMD as total CSA was similar in those with and without HIV. In contrast, only in WLWH did we see any indication of compromised torsional (pSSI) bone strength which reflects female cortical compartment impairments (i.e. lower cortical vBMD and cortical thickness). Interestingly, these findings were consistent between load bearing and non-load bearing limbs and were largely independent of weight. Overall, our data suggest that post-menopausal HIV-related bone impairments may represent a worsening of the natural age-related pattern of female bone loss; findings are consistent with, but extend our previous findings of poorer cortical vBMD by menopause stage in midlife South African WLWH^22^ and by age in midlife Zimbabwean WLWH.^21^ However, in the present study we found adjustment for menopause made little difference, likely because most women were postmenopausal.

Internationally, few studies have reported on the compartment-specific influence of HIV and bone in both men and women recruited from the same location using common recruitment parameters. This means that although data within-sex exist, comparisons between studies may be confounded by a host of factors. Studies from high income settings that have reported compartment-specific differences between PLWH and HIV-negative controls have largely used central QCT which involves far greater ionizing radiation exposure or high-resolution pQCT (HR-pQCT), of which there are few globally in clinical settings. Our findings are consistent with a small US QCT study of PLWH aged 50-70 years (n = 66, 61% PLWH) that reported poorer trabecular vBMD and architecture at the femoral neck, trochanter and total hip, relative to controls without HIV.^42^ Similarly, a male only South Korean QCT study documented midlife HIV-related cortical-impairments present at the proximal femur, the site of fragility fracture of the hip.^43^ Interestingly, we did not observe cortical impairment in the peripheral skeletons of our older MLWH, though this may be attributable to the lower resolution of pQCT or possibly because bone losses may initially present in the axial rather than the appendicular skeleton. Several QCT studies have reported compartment-specific impairments in younger WLWH,^44^ midlife WLWH^40^^,^^45^ or postmenopausal WLWH^46^ relative to HIV-negative women. A small Swiss study (n = 66, 33% WLWH) of premenopausal WLWH reported micro-architectural impairment in (first generation) HR-pQCT assessed distal tibia trabecular vBMD (-14.1 %) and distal radius cortical vBMD (-3%)^44^ comparable to those documented in the present study and our previous midlife study.^21^ MacDonald et al. in a Canadian study also reported poorer (first generation) HR-pQCT trabecular vBMD in midlife WLWH relative to controls.^45^ Finally, Yin et al in a US study of postmenopausal WLWH and HIV-negative controls (n =  106, 43% WLWH) found lower cortical CSA and thickness (11-12%) in WLWH relative to controls.^46^ Notable differences between these studies and our study include participant use of methadone at the time of recruitment and/or high rates of historic illicit drug use, a wider availability of non-TDF ART regimens, racially diverse samples, and the use of first-generation HR-pQCT which images the distal radius/tibia where the cortex is relatively thin to obtain its cortical bone parameters.

Although age at HIV diagnosis, years since diagnosis, and ART duration were similar by sex, associations between time since diagnosis and bone were primarily present in women (after adjustment for ART duration). These findings are comparable to previous observations in somewhat younger mid-life Zimbabwean women.^21^ In the current study, PLWH were relatively homogenous regarding TDF use (>90%) and viral suppression (>80%), which may explain the limited associations between these exposures and bone outcomes. Given TDF is a component of first-line ART,^47^ thus those not on TDF would be expected to be less healthy, having failed first line therapy; we have previously encountered similar in this setting.^21^ However, our finding of tibial cortical vBMD impairment associated with TDF-use in men fits with the previously described bone effects of TDF,^48^^,^^49^ despite international data regarding compartment-specific effects of TDF-based regimens are somewhat conflicting.^44^^,^^45^ Interestingly, a US study (n = 43) of PLWH reported that only TDF alongside a protease inhibitor (PI) was associated with poorer bone microstructure at the distal tibia.^50^ This was supported by QCT findings at the proximal femur in South Korean MLWH^43^. Given the very limited use of PIs (n =  6, <3%) in our population this could not be explored. Our finding of lower radial pQCT parameters in women with good viral suppression appears counter intuitive but aligns with similar findings in Canadian WLWH^45^^,^^51^^,^^52^ and may be attributable to the fact that ART initiation leads to T-cell repopulation and the secretion of osteoclastogenic cytokines (including RANKL and tumor necrosis factor (TNF)).

### Strengths and limitations

This study has strengths including its large sample size, to date the largest pQCT study on the continent. These are unique data from an under-represented population, older African PLWH, with a similar comparator group drawn from the same population-based community sample recruited using GIS-mapping. Findings have some generalizability to the wider Southern African region where the prevalence of chronic ART-controlled HIV in older PLWH remains high and ART regimes are similar.^53^ Limitations include the study design being cross-sectional so temporal directions of association cannot be established and recall bias may have limited detection of self-reported exposures. While fracture history was in part captured by self-report which is subject to detection and recall biases, we were able to independently assess vertebral fractures using later DXA. As fractures are rare events, analyses were under-powered and purely exploratory. pQCT-derived strength indices are known to correlate highly with vBMD and may not fully represent the mechanical properties of bone due to the limited resolution of pQCT compared to higher resolution techniques. ART data were insufficient to determine effects of sequential/switching regimes.

## Conclusions

Older Zimbabweans living with HIV have substantially poorer trabecular vBMD, whether male or female. Whilst, HIV-related cortical compartment impairments were only evident in women, who were mostly post-menopausal. This study adds to the growing literature from Southern Africa highlighting the urgent need to prioritize bone health across the lifecourse for PLWH. Compartment and sex-specific impairments relating to ART regimen and viral suppression warrant further investigation; their influence on bone is complex and cannot be fully disentangled in cross-sectional analyses. Equally, although clear impairments in pQCT measures were evident in those who had had a prior MOF, longitudinal studies are needed to fully elucidate the relationships between trabecular and cortical architecture and future site-specific fracture risk.

## Supplementary Material

MS_ASBMR-25090748_Supplementary_materials_2026_05_06_zjag082

STROBE_Strengthening_the_Reporting_of_Observational_Studies_in_Epidemiology

## Data Availability

The data underlying this article will be shared on reasonable request to the senior author.
