# Supplementary material for: Sex-specific bone architectural deficits among older adults living with HIV: a cross-sectional study from Zimbabwe
Source: J Bone Miner Res. 2026 May 12;41(8):848–58. doi: 10.1093/jbmr/zjag082 (PMC13421066; doi:10.1093/jbmr/zjag082)

**Supplementary materials**

Table S1: pQCT bone outcomes of Zimbabwean men and women aged 40 years and older by HIV status

| **HIV status** | **HIV-negative men** | **MLWH** |  | **HIV-negative women** | **WLWH** |  |
| --- | --- | --- | --- | --- | --- | --- |
| *Radius 4%* | n = 423 | n = 106 | p-value | n = 443 | n = 115 | p-value |
| Total vBMD (mg/cm^3^) | 336 (66) | 325 (57) | 0.071 | 292 (71) | 298 (62) | 0.348 |
| Trabecular vBMD (mg/cm^3^) | 191 (49) | 173 (51) | 0.002 | 146 (46) | 144 (48) | 0.774 |
| Total CSA (mm^2^) | 416 (64) | 423 (63) | 0.347 | 345 (60) | 334 (50) | 0.050 |
| BSIc (g^2^/cm^4^) | 0.48 (0.17) | 0.45 (0.15) | 0.139 | 0.30 (0.13) | 0.30 (0.11) | 0.807 |
| *Radius 33%* | n = 421 | n = 106 | p-value | n = 437 | n = 114 | p-value |
| Cortical vBMD (mg/cm^3^) | 1210 (40) | 1220 (30) | 0.022 | 1180 (60) | 1190 (50) | 0.087 |
| Cortical thickness (mm) | 2.79 (0.39) | 2.81 (0.33) | 0.582 | 2.16 (0.49) | 2.25 (0.42) | 0.064 |
| Total CSA (mm^2^) | 148 (19) | 148 (19) | 0.944 | 116 (16) | 114 (16) | 0.249 |
| pSSI (mm^3^) | 362 (67) | 364 (63) | 0.821 | 238 (51) | 238 (51) | 0.938 |
| *Tibia 4%* | n = 424 | n = 107 | p-value | n = 446 | n = 116 | p-value |
| Total vBMD (mg/cm^3^) | 278 (55) | 261 (47) | 0.001 | 260 (53) | 261 (49) | 0.806 |
| Trabecular vBMD (mg/cm^3^) | 200 (48) | 181 (43) | <0.001 | 187 (47) | 182 (41) | 0.334 |
| Total CSA (mm^2^) | 1200 (160) | 1220 (150) | 0.203 | 984 (119) | 986 (114) | 0.886 |
| BSIc (g^2^/cm^4^) | 0.95 (0.35) | 0.85 (0.30) | 0.003 | 0.69 (0.26) | 0.68 (0.23) | 0.997 |
| *Tibia 38%* | n = 424 | n = 107 | p-value | n = 445 | n = 116 | p-value |
| Cortical vBMD (mg/cm^3^) | 1200 (40) | 1210 (30) | 0.297 | 1170 (50) | 1180 (50) | 0.620 |
| Cortical thickness (mm) | 5.15 (0.74) | 5.13 (0.67) | 0.772 | 4.04 (0.88) | 4.11 (0.78) | 0.446 |
| Total CSA (mm^2^) | 497 (67) | 499 (58) | 0.799 | 405 (50) | 408 (43) | 0.547 |
| pSSI (mm^3^) | 2270 (420) | 2290 (400) | 0.697 | 1570 (320) | 1590 (280) | 0.573 |
| MLWH = men living with HIV, WLWH = women living with HIV, vBMD = volumetric bone mineral density, CSA = cross-sectional area, BSIc = bone strength index of compression, pSSI = polar stress-strain index P-value from independent samples unpaired t-test | | | | | | |

| Supplementary Table S2. pQCT parameters by MOF status in older Zimbabwean adults | | | | | | |
| --- | --- | --- | --- | --- | --- | --- |
| Original units | Unadjusted | | | Adjusted for age, sex, and wealth index | | |
| *Radius* | No MOF mean (95% CI) | Prior MOF mean (95% CI) | Mean difference (95% CI) | No MOF mean (95% CI) | Prior MOF mean (95% CI) | Mean difference (95% CI) |
| Total vBMD (mg/cm^3^) | 316.89  (312.56, 321.22) | 277.87  (264.77, 290.97) | -39.02  (-52.82, -25.22) | 315.81  (312.07, 319.55) | 293.20  (281.77, 304.63) | -22.61  (-34.66, -10.56) |
| Trabecular vBMD (mg/cm^3^) | 167.98  (164.71, 171.25) | 145.60  (135.71, 155.50) | -22.38  (-32.80, -11.96) | 167.86  (164.95, 170.77) | 152.34  (143.45, 161.23) | -15.52  (-24.89, -6.15) |
| Total CSA (mm^2^) | 379.41  (374.92, 383.89) | 377.23  (363.66, 390.80) | -2.17  (-16.47, 12.12) | 380.35  (376.52, 384.17) | 378.69  (367.00, 390.38) | -1.66  (-13.98, 10.67) |
| BSIc (g^2^/cm^4^) | 0.39 (0.38, 0.40) | 0.30 (0.27, 0.33) | -0.09 (-0.12, -0.06) | 0.39 (0.38, 0.40) | 0.34 (0.31, 0.36) | -0.05 (-0.08, -0.03) |
| Cortical vBMD (mg/cm^3^) | 1200.97  (1197.80, 1204.14) | 1173.17  (1163.63, 1182.71) | -27.80  (-37.85, -17.75) | 1199.82  (1197.26, 1202.39) | 1186.92  (1179.10, 1194.74) | -12.90  (-21.16, -4.65) |
| Cortical thickness (mm) | 2.51 (2.48, 2.54) | 2.21 (2.11, 2.31) | -0.31 (-0.41, -0.20) | 2.51 (2.48, 2.53) | 2.33 (2.26, 2.40) | -0.17 (-0.25, -0.10) |
| Total CSA (mm^2^) | 131.67  (130.16, 133.17) | 128.40  (123.87, 132.93) | -3.27 (-8.04, 1.50) | 131.97  (130.86, 133.08) | 129.22  (125.84, 132.60) | -2.76 (-6.32, 0.81) |
| pSSI (mm^3^) | 301.45  (296.06, 306.83) | 275.14  (258.91, 291.37) | -26.31  (-43.41, -9.21) | 301.98  (298.32, 305.65) | 284.20  (273.03, 295.36) | -17.78  (-29.56, -6.01) |
| *Tibia* |  |  |  |  |  |  |
| Total vBMD (mg/cm^3^) | 270.50  (267.21, 273.80) | 237.28  (227.32, 247.24) | -33.22  (-43.71, -22.73) | 269.73  (266.67, 272.79) | 246.25  (236.91, 255.59) | -23.48  (-33.33, -13.64) |
| Trabecular vBMD (mg/cm^3^) | 193.45  (190.55, 196.35) | 168.23  (159.47, 176.98) | -25.22  (-34.44, -16.00) | 193.16  (190.34, 195.99) | 172.20  (163.58, 180.83) | -20.96  (-30.06, -11.87) |
| Total CSA (mm^2^) | 1094.12  (1083.09, 1105.16) | 1069.00  (1035.67, 1102.33) | -25.12  (-60.23, 9.99) | 1095.96  (1087.33, 1104.58) | 1083.79  (1057.49, 1110.10) | -12.16  (-39.90, 15.57) |
| BSIc (g^2^/cm^4^) | 0.82 (0.80, 0.84) | 0.63 (0.57, 0.69) | -0.19 (-0.25, -0.12) | 0.82 (0.80, 0.84) | 0.69 (0.64, 0.75) | -0.13 (-0.18, -0.07) |
| Cortical vBMD (mg/cm^3^) | 1189.98  (1186.95, 1193.01) | 1164.69  (1155.55, 1173.83) | -25.29  (-34.91, -15.66) | 1189.21  (1186.63, 1191.80) | 1175.68  (1167.81, 1183.56) | -13.53  (-21.84, -5.23) |
| Cortical thickness (mm) | 4.63 (4.57, 4.69) | 4.14 (3.96, 4.32) | -0.50 (-0.69, -0.31) | 4.63 (4.59, 4.68) | 4.31 (4.16, 4.45) | -0.33 (-0.48, -0.18) |
| Total CSA (mm^2^) | 451.07  (446.49, 455.65) | 440.75  (426.93, 454.57) | -10.32  (-24.88, 4.24) | 451.80  (448.23, 455.36) | 446.86  (436.00, 457.72) | -4.94  (-16.39, 6.51) |
| pSSI (mm^3^) | 1931.04  (1899.42, 1962.65) | 1773.15  (1677.72, 1868.58) | -157.88  (-258.42, -57.35) | 1933.66  (1911.38, 1955.94) | 1845.61  (1777.69, 1913.53) | -88.05  (-159.68, -16.43) |
| vBMD = volumetric bone mineral density, CSA = cross-sectional area, BSIc = bone strength index of compression, pSSI = polar stress-strain index | | | | | | |

| Supplementary Table S3. pQCT parameters by MOF status in older Zimbabwean adults living with HIV | | | | | | |
| --- | --- | --- | --- | --- | --- | --- |
|  | Unadjusted | | | Adjusted for age, sex, and wealth index | | |
|  | No MOF  mean (95% CI) | Prior MOF  mean (95% CI) | Mean difference  (95% CI) | No MOF  mean (95% CI) | Prior MOF  mean (95% CI) | Mean difference  (95% CI) |
| Radius total vBMD (mg/cm^3^) | 311.76  (303.32, 320.20) | 301.88  (273.54, 330.23) | -9.88  (-39.45, 19.70) | 311.60  (303.88, 319.32) | 310.35  (284.33, 336.38) | -1.24  (-28.40, 25.91) |
| Radius trabecular vBMD (mg/cm^3^) | 159.27  (152.21, 166.34) | 146.04  (122.31, 169.77) | -13.23  (-37.99, 11.53) | 159.51  (152.83, 166.19) | 150.60  (128.08, 173.12) | -8.91  (-32.41, 14.59) |
| Radius 4% total CSA (mm^2^) | 378.59  (368.67, 388.51) | 355.38  (322.07, 388.68) | -23.21  (-57.96, 11.53) | 380.19  (372.34, 388.05) | 359.26  (332.79, 385.73) | -20.93  (-48.55, 6.69) |
| Radius BSIc (g^2^/cm^4^) | 0.38  (0.36, 0.40) | 0.33  (0.27, 0.40) | -0.04  (-0.11, 0.03) | 0.38  (0.36, 0.40) | 0.36  (0.30, 0.41) | -0.02  (-0.08, 0.04) |
| Radius cortical vBMD (mg/cm^3^) | 1208.24  (1201.85, 1214.63) | 1174.11  (1152.70, 1195.51) | -34.14  (-56.47, -11.80) | 1208.06  (1202.80, 1213.31) | 1182.67  (1164.99, 1200.34) | -25.39  (-43.84, -6.94) |
| Radius cortical thickness (mm) | 2.55  (2.48, 2.61) | 2.21  (2.00, 2.43) | -0.34  (-0.56, -0.11) | 2.55  (2.51, 2.60) | 2.30  (2.15, 2.45) | -0.25  (-0.40, -0.09) |
| Radius 33% total CSA (mm^2^) | 131.00  (127.67, 134.34) | 123.39  (112.21, 134.57) | -7.61  (-19.28, 4.05) | 131.56  (129.18, 133.93) | 124.69  (116.71, 132.67) | -6.87  (-15.19, 1.46) |
| Radius pSSI (mm^3^) | 302.44  (290.77, 314.11) | 257.16  (218.06, 296.27) | -45.28  (-86.09, -4.47) | 304.19  (296.38, 312.01) | 265.23  (238.93, 291.52) | -38.97  (-66.41, -11.53) |
| Tibia total vBMD (mg/cm^3^) | 262.52  (255.89, 269.15) | 245.36  (223.64, 267.08) | -17.16  (-39.87, 5.55) | 262.14  (255.77, 268.50) | 249.26  (228.32, 270.19) | -12.88  (-34.77, 9.00) |
| Tibia trabecular vBMD (mg/cm^3^) | 182.83  (177.04, 188.62) | 169.23  (150.26, 188.21) | -13.60  (-33.43, 6.24) | 182.61  (176.88, 188.35) | 171.09  (152.21, 189.96) | -11.53  (-31.26, 8.20) |
| Tibia 4% total CSA (mm^2^) | 1101.50  (1077.29, 1125.72) | 1071.04  (991.70, 1150.38) | -30.46  (-113.42, 52.49) | 1104.93  (1086.94, 1122.92) | 1089.65  (1030.47, 1148.83) | -15.28  (-77.15, 46.58) |
| Tibia BSIc (g^2^/cm^4^) | 0.77  (0.73, 0.81) | 0.67  (0.55, 0.80) | -0.10  (-0.23, 0.03) | 0.77  (0.74, 0.81) | 0.70  (0.59, 0.82) | -0.07  (-0.19, 0.05) |
| Tibia cortical vBMD (mg/cm^3^) | 1191.89  (1185.34, 1198.45) | 1169.04  (1147.56, 1190.53) | -22.85  (-45.32, -0.39) | 1191.76  (1186.42, 1197.11) | 1177.56  (1159.97, 1195.16) | -14.20  (-32.59, 4.20) |
| Tibia cortical thickness (mm) | 4.62  (4.50, 4.74) | 4.34  (3.93, 4.74) | -0.28  (-0.70, 0.13) | 4.63  (4.53, 4.72) | 4.48  (4.16, 4.79) | -0.15  (-0.48, 0.17) |
| Tibia 38% total CSA (mm^2^) | 452.31  (442.92, 461.70) | 439.84  (409.07, 470.61) | -12.47  (-44.64, 19.71) | 453.68  (446.67, 460.69) | 446.50  (423.45, 469.55) | -7.18  (-31.28, 16.91) |
| Tibia pSSI (mm^3^) | 1938.35  (1871.28, 2005.42) | 1775.15  (1555.37, 1994.92) | -163.20  (-392.98, 66.58) | 1946.97  (1901.20, 1992.74) | 1846.88  (1696.31, 1997.45) | -100.09  (-257.49, 57.32) |
| vBMD = volumetric bone mineral density, CSA = cross-sectional area, BSIc = bone strength index of compression, pSSI = polar stress-strain index | | | | | | |

| Supplementary Table S4. pQCT parameters by MOF status in older Zimbabwean adults without HIV | | | | | | |
| --- | --- | --- | --- | --- | --- | --- |
|  | Unadjusted | | | Adjusted for age, sex, and wealth index | | |
|  | No MOF mean  (95% CI) | Prior MOF mean (95% CI) | Mean difference (95% CI) | No MOF mean (95% CI) | Prior MOF mean (95% CI) | Mean difference (95% CI) |
| Radius total vBMD (mg/cm^3^) | 318.23  (313.24, 323.22) | 273.01  (258.26, 287.76) | -45.22  (-60.79, -29.64) | 316.80  (312.61, 320.99) | 290.38  (277.85, 302.92) | -26.41  (-39.66, -13.17) |
| Radius trabecular vBMD (mg/cm^3^) | 170.26  (166.58, 173.94) | 145.51  (134.64, 156.39) | -24.74  (-36.23, -13.26) | 169.92  (166.73, 173.10) | 153.50  (143.98, 163.01) | -16.42  (-26.48, -6.36) |
| Radius 4% total CSA (mm^2^) | 379.62  (374.59, 384.66) | 381.65  (366.78, 396.53) | 2.03  (-13.67, 17.74) | 380.40  (376.04, 384.77) | 382.77  (369.73, 395.81) | 2.37  (-11.42, 16.15) |
| Radius BSIc (g^2^/cm^4^) | 0.40  (0.38, 0.41) | 0.30  (0.26, 0.33) | -0.10  (-0.14, -0.06) | 0.39  (0.38, 0.40) | 0.34  (0.31, 0.36) | -0.06  (-0.08, -0.03) |
| Radius cortical vBMD (mg/cm^3^) | 1199.06  (1195.44, 1202.69) | 1172.98  (1162.32, 1183.64) | -26.08  (-37.34, -14.82) | 1197.68  (1194.76, 1200.60) | 1187.63  (1178.93, 1196.32) | -10.05  (-19.25, -0.86) |
| Radius cortical thickness (mm) | 2.50  (2.46, 2.54) | 2.21  (2.09, 2.32) | -0.30  (-0.41, -0.18) | 2.49  (2.47, 2.52) | 2.34  (2.26, 2.42) | -0.15  (-0.24, -0.07) |
| Radius 33% total CSA (mm^2^) | 131.84  (130.15, 133.53) | 129.41  (124.45, 134.37) | -2.43  (-7.67, 2.81) | 132.08  (130.82, 133.34) | 130.21  (126.46, 133.95) | -1.87  (-5.83, 2.09) |
| Radius pSSI (mm^3^) | 301.18  (295.10, 307.27) | 278.77  (260.89, 296.66) | -22.41  (-41.30, -3.52) | 301.38  (297.22, 305.53) | 288.24  (275.88, 300.60) | -13.14  (-26.21, -0.06) |
| Tibia total vBMD (mg/cm^3^) | 272.59  (268.81, 276.36) | 235.56  (224.38, 246.74) | -37.03  (-48.83, -25.23) | 271.58  (268.17, 275.00) | 246.49  (236.26, 256.72) | -25.10  (-35.91, -14.29) |
| Tibia trabecular vBMD (mg/cm^3^) | 196.22  (192.91, 199.53) | 168.01  (158.21, 177.82) | -28.21  (-38.55, -17.86) | 195.79  (192.61, 198.98) | 173.34  (163.79, 182.88) | -22.46  (-32.54, -12.37) |
| Tibia 4% total CSA (mm^2^) | 1092.19  (1079.77, 1104.62) | 1068.57  (1031.76, 1105.38) | -23.63  (-62.48, 15.23) | 1093.68  (1083.85, 1103.51) | 1082.23  (1052.76, 1111.69) | -11.45  (-42.59, 19.68) |
| Tibia BSIc (g^2^/cm^4^) | 0.83  (0.81, 0.86) | 0.63  (0.56, 0.69) | -0.21  (-0.28, -0.14) | 0.83  (0.81, 0.85) | 0.70  (0.64, 0.75) | -0.13  (-0.20, -0.07) |
| Tibia cortical vBMD (mg/cm^3^) | 1189.48  (1186.06, 1192.90) | 1163.76  (1153.64, 1173.88) | -25.72  (-36.40, -15.03) | 1188.54  (1185.63, 1191.45) | 1175.40  (1166.68, 1184.12) | -13.13  (-22.35, -3.92) |
| Tibia cortical thickness (mm) | 4.64  (4.57, 4.71) | 4.10  (3.89, 4.30) | -0.54  (-0.76, -0.33) | 4.63  (4.58, 4.69) | 4.27  (4.11, 4.43) | -0.36  (-0.53, -0.19) |
| Tibia 38% total CSA (mm^2^) | 450.75  (445.51, 455.98) | 440.94  (425.44, 456.44) | -9.81  (-26.17, 6.56) | 451.30  (447.18, 455.41) | 446.96  (434.65, 459.28) | -4.33  (-17.35, 8.68) |
| Tibia pSSI (mm^3^) | 1929.12  (1893.22, 1965.02) | 1772.73  (1666.45, 1879.00) | -156.40  (-268.57, -44.22) | 1930.08  (1904.61, 1955.55) | 1846.25  (1769.99, 1922.51) | -83.83  (-164.43, -3.24) |
| vBMD = volumetric bone mineral density, CSA = cross-sectional area, BSIc = bone strength index of compression, pSSI = polar stress-strain index | | | | | | |

| Supplementary Table S5. pQCT parameters by MOF status in older Zimbabwean adults living with HIV, further adjusted for HIV specific factors | | | | | | |
| --- | --- | --- | --- | --- | --- | --- |
|  | Adjusted for age, sex, wealth index **and time since HIV diagnosis** | | | Adjusted for age, sex, wealth index, time since HIV diagnosis **and ART duration** | | |
|  | No MOF  mean (95% CI) | Prior MOF  mean (95% CI) | Mean difference (95% CI) | No MOF  mean (95% CI) | Prior MOF  mean (95% CI) | Mean difference (95% CI) |
| Radius total vBMD (mg/cm^3^) | 311.60  (303.93, 319.27) | 316.04  (289.44, 342.64) | 4.44  (-23.26, 32.14) | 311.25  (303.07, 319.42) | 317.97  (289.36, 346.59) | 6.73  (-23.07, 36.52) |
| Radius trabecular vBMD (mg/cm^3^) | 159.47  (152.82, 166.13) | 155.25  (132.19, 178.32) | -4.22  (-28.24, 19.80) | 159.54  (152.34, 166.74) | 155.88  (130.68, 181.08) | -3.66  (-29.89, 22.58) |
| Radius 4% total CSA (mm^2^) | 380.24  (372.40, 388.08) | 356.21  (329.02, 383.40) | -24.03  (-52.34, 4.28) | 378.81  (370.45, 387.17) | 350.06  (320.79, 379.33) | -28.75  (-59.23, 1.72) |
| Radius BSIc (g^2^/cm^4^) | 0.38  (0.36, 0.40) | 0.37  (0.31, 0.42) | -0.01  (-0.07, 0.05) | 0.38  (0.36, 0.39) | 0.36  (0.29, 0.42) | -0.02  (-0.08, 0.05) |
| Radius cortical vBMD (mg/cm^3^) | 1208.13  (1202.85, 1213.41) | 1182.70  (1164.43, 1200.97) | -25.43  (-44.46, -6.41) | 1207.26  (1201.53, 1212.99) | 1186.79  (1166.79, 1206.79) | -20.48  (-41.31, 0.36) |
| Radius cortical thickness (mm) | 2.55  (2.51, 2.60) | 2.31  (2.15, 2.46) | -0.25  (-0.41, -0.08) | 2.54  (2.50, 2.59) | 2.32  (2.15, 2.49) | -0.22  (-0.40, -0.05) |
| Radius 33% total CSA (mm^2^) | 131.56  (129.19, 133.94) | 124.99  (116.78, 133.20) | -6.57  (-15.12, 1.98) | 130.92  (128.47, 133.36) | 124.22  (115.68, 132.75) | -6.70  (-15.59, 2.19) |
| Radius pSSI (mm^3^) | 304.23  (296.39, 312.07) | 265.61  (238.47, 292.74) | -38.63  (-66.88, -10.37) | 302.11  (294.16, 310.05) | 264.72  (236.98, 292.46) | -37.38  (-66.27, -8.49) |
| Tibia total vBMD (mg/cm^3^) | 262.11  (255.84, 268.37) | 256.08  (234.90, 277.26) | -6.03  (-28.12, 16.07) | 262.28  (255.61, 268.94) | 247.41  (224.76, 270.06) | -14.86  (-38.50, 8.77) |
| Tibia trabecular vBMD (mg/cm^3^) | 182.57  (176.92, 188.22) | 177.32  (158.23, 196.42) | -5.25  (-25.17, 14.67) | 182.08  (176.03, 188.12) | 170.62  (150.06, 191.18) | -11.46  (-32.91, 10.00) |
| Tibia 4% total CSA (mm^2^) | 1105.12  (1087.18, 1123.06) | 1085.46  (1024.84, 1146.09) | -19.65  (-82.90, 43.60) | 1101.91  (1082.71, 1121.11) | 1095.21  (1029.94, 1160.49) | -6.70  (-74.81, 61.42) |
| Tibia BSIc (g^2^/cm^4^) | 0.77  (0.74, 0.81) | 0.73  (0.61, 0.85) | -0.04  (-0.16, 0.08) | 0.77  (0.73, 0.81) | 0.68  (0.55, 0.80) | -0.09  (-0.22, 0.04) |
| Tibia cortical vBMD (mg/cm^3^) | 1191.84  (1186.48, 1197.19) | 1180.35  (1162.27, 1198.43) | -11.49  (-30.35, 7.37) | 1191.66  (1185.84, 1197.48) | 1177.06  (1157.27, 1196.84) | -14.60  (-35.25, 6.04) |
| Tibia cortical thickness (mm) | 4.63  (4.53, 4.72) | 4.56  (4.24, 4.87) | -0.07  (-0.40, 0.26) | 4.63  (4.53, 4.73) | 4.52  (4.18, 4.86) | -0.11  (-0.47, 0.24) |
| Tibia 38% total CSA (mm^2^) | 453.74  (446.79, 460.69) | 450.00  (426.51, 473.48) | -3.74  (-28.24, 20.76) | 454.28  (447.13, 461.43) | 446.10  (421.80, 470.40) | -8.18  (-33.53, 17.17) |
| Tibia pSSI (mm^3^) | 1947.23  (1901.56, 1992.90) | 1872.44  (1718.12, 2026.75) | -74.79  (-235.79, 86.20) | 1949.82  (1902.41, 1997.23) | 1838.58  (1677.40, 1999.75) | -111.25  (-279.43, 56.94) |
| vBMD = volumetric bone mineral density, CSA = cross-sectional area, BSIc = bone strength index of compression, pSSI = polar stress-strain index | | | | | | |

Figure S1. Participant flow chart describing household-based identification and recruitment of study participants.


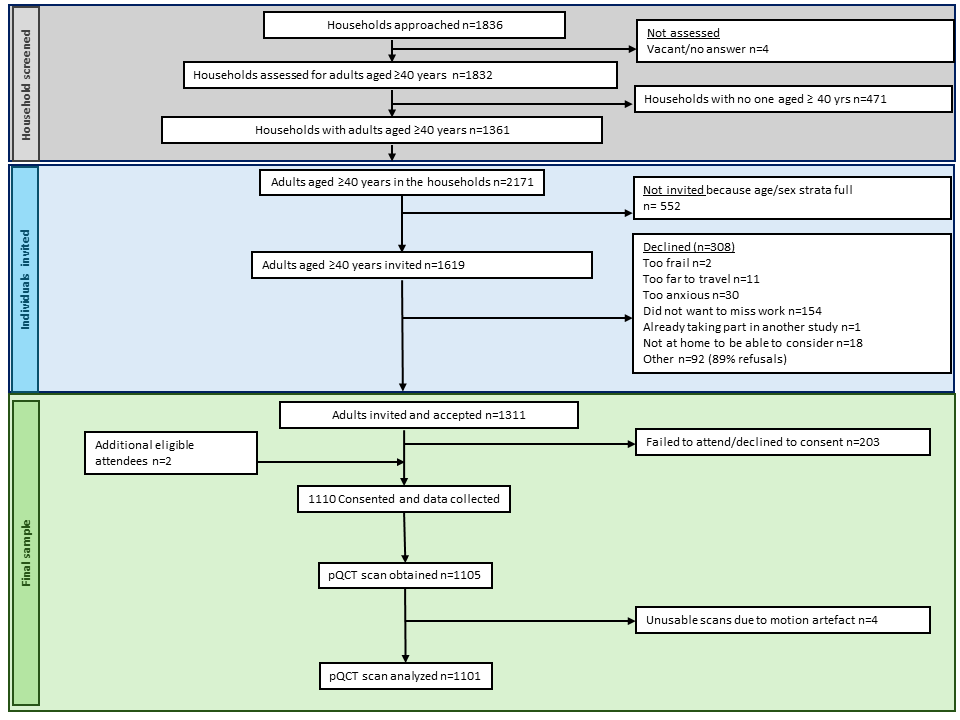


Figure S2A. pQCT outcomes at the distal radius in older Zimbabweans men and women. P-values express the relationship between each pQCT outcome and age within-sex and HIV strata. i) total vBMD, ii) trabecular vBMD, iii) total CSA, iv) BSIc. vBMD, volumetric BMD; CSA, cross-sectional area; BSIc, bone strength index of compression


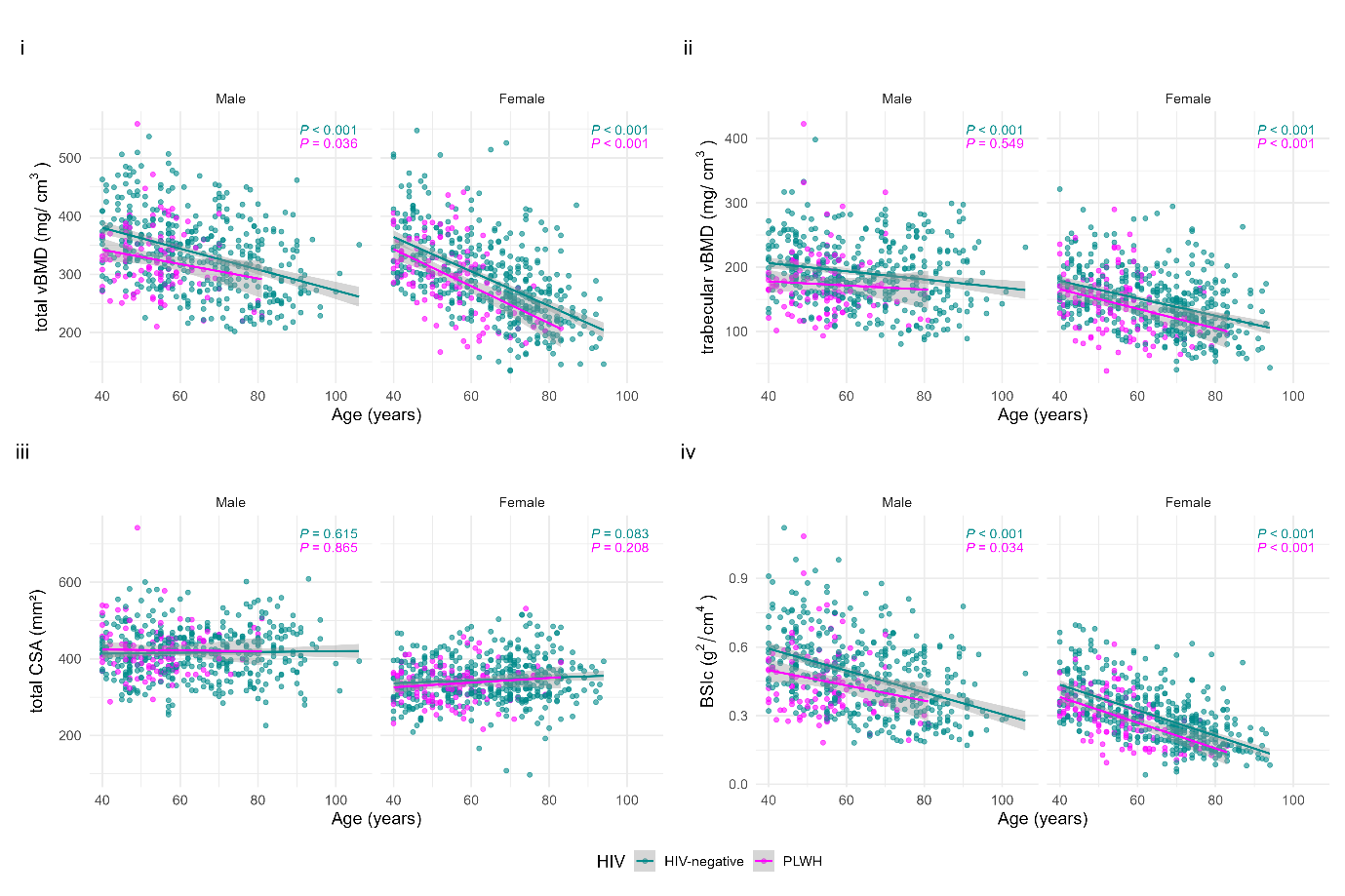


Figure S2B. pQCT outcomes at the proximal radius in older Zimbabweans men and women. P-values express the linear relationship between each pQCT outcome and age within-sex and HIV strata. i) cortical vBMD, ii) cortical thickness, iii) total CSA, iv) SSI. vBMD, volumetric BMD; CSA, cross-sectional area; SSI, stress–strain index.


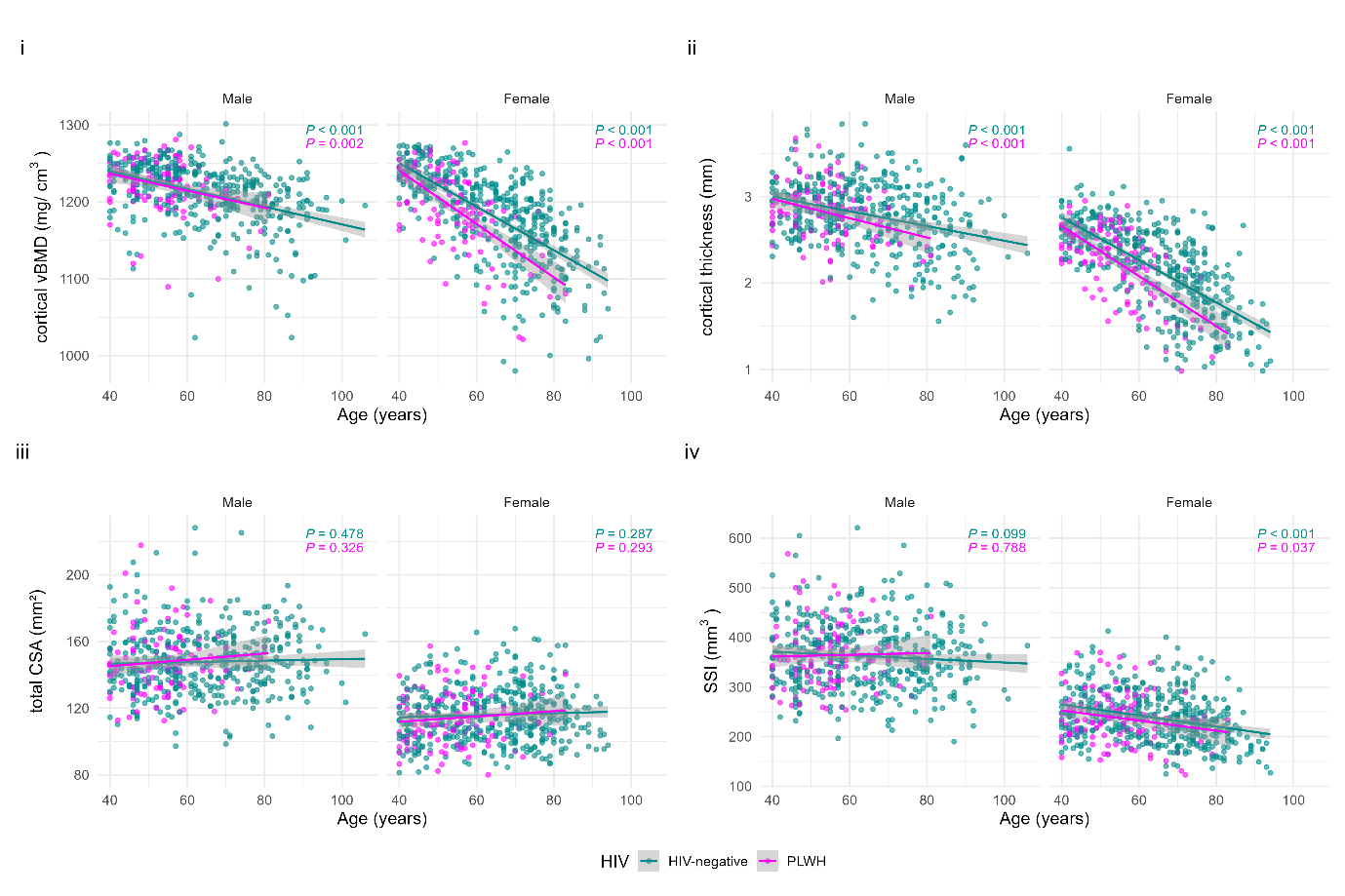


**Figure S3A.** pQCT outcomes at the distal tibia in older Zimbabweans men and women. P-values express the linear relationship between each pQCT outcome and age within-sex and HIV strata. i) total vBMD, ii) trabecular vBMD, iii) total CSA, iv) BSIc. vBMD, volumetric BMD; CSA, cross-sectional area; BSIc, bone strength index of compression


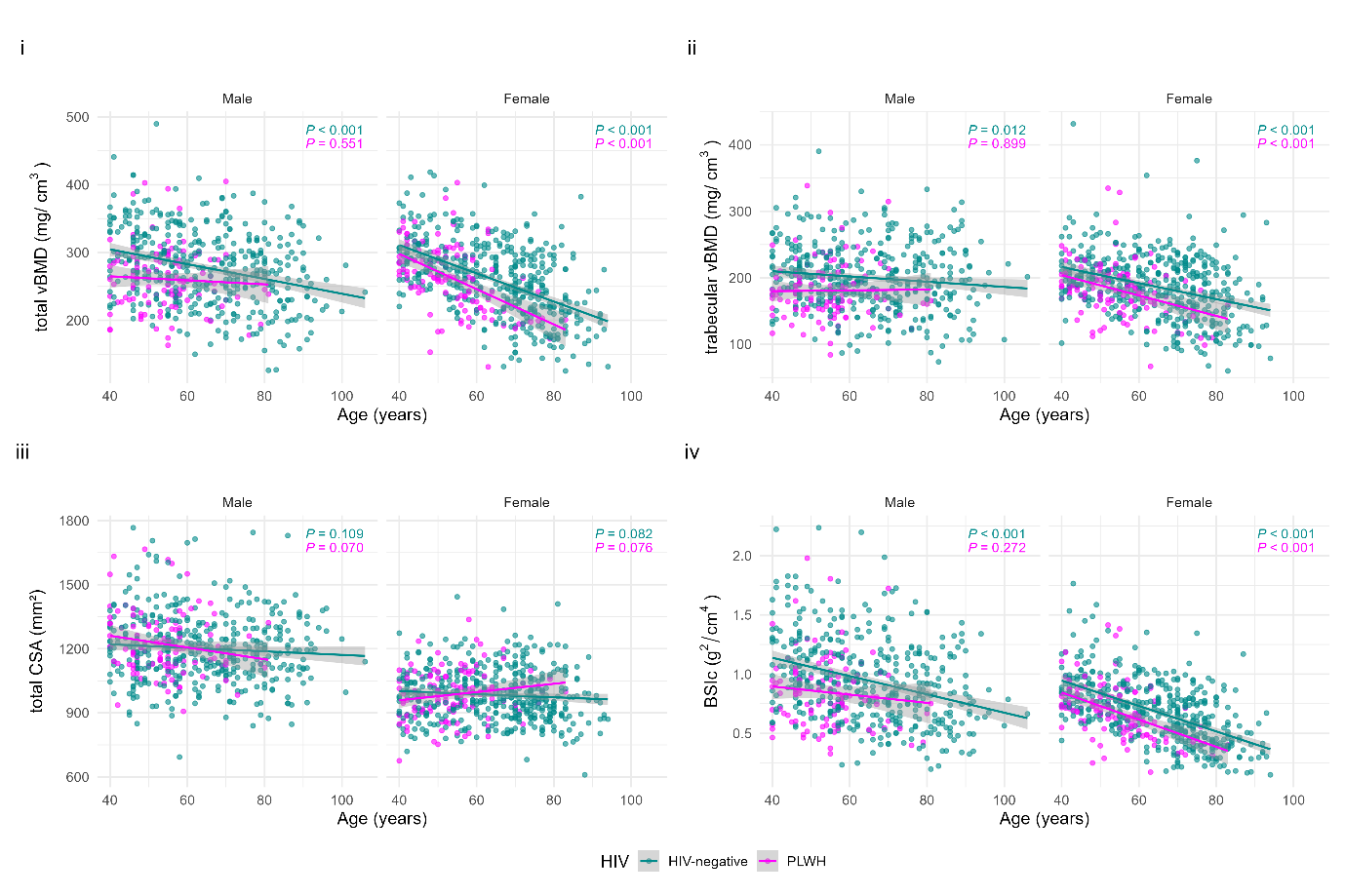


**Figure S3B.** pQCT outcomes at the proximal tibia in older Zimbabweans men and women. P-values express the linear relationship between each pQCT outcome and age within-sex and HIV strata. i) cortical vBMD, ii) cortical thickness, iii) total CSA, iv) SSI. vBMD, volumetric BMD; CSA, cross-sectional area; SSI, stress–strain index.


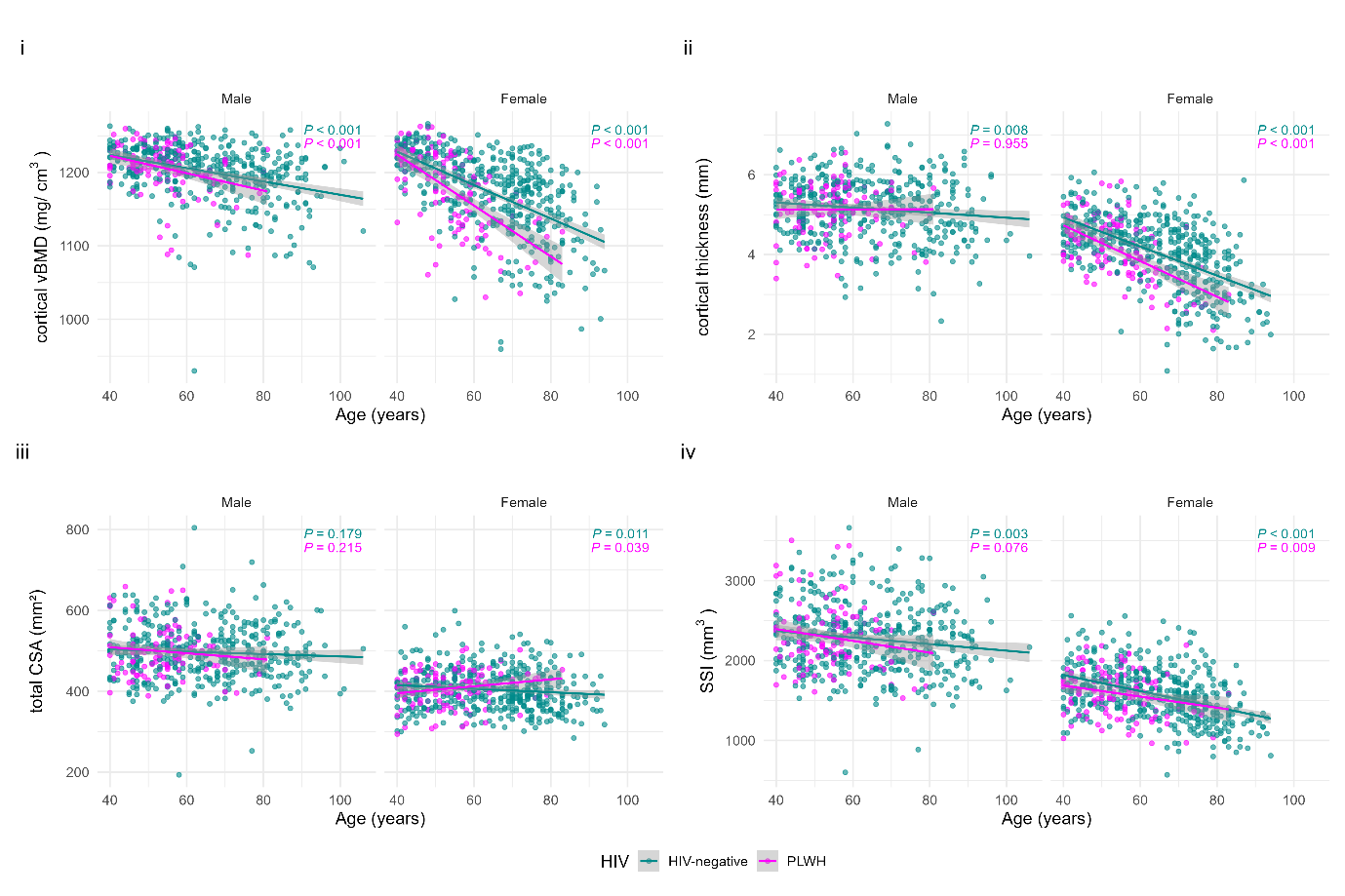


**Figure S4.**  Estimates of symmetric percentage differences in pQCT parameters at the tibia by HIV status in A) men and B) women. Model 1 adjusted for age (yr) and wealth index; model 2 adjusted for age (yr), wealth index, height (m), and weight (kg). Tests for sex*HIV interactions were performed with no interactions p<0.05 detected. Abbreviations: vBMD, volumetric BMD; CSA, cross-sectional area; BSIc, bone strength index of compression; pSSI, polar stress–strain index.


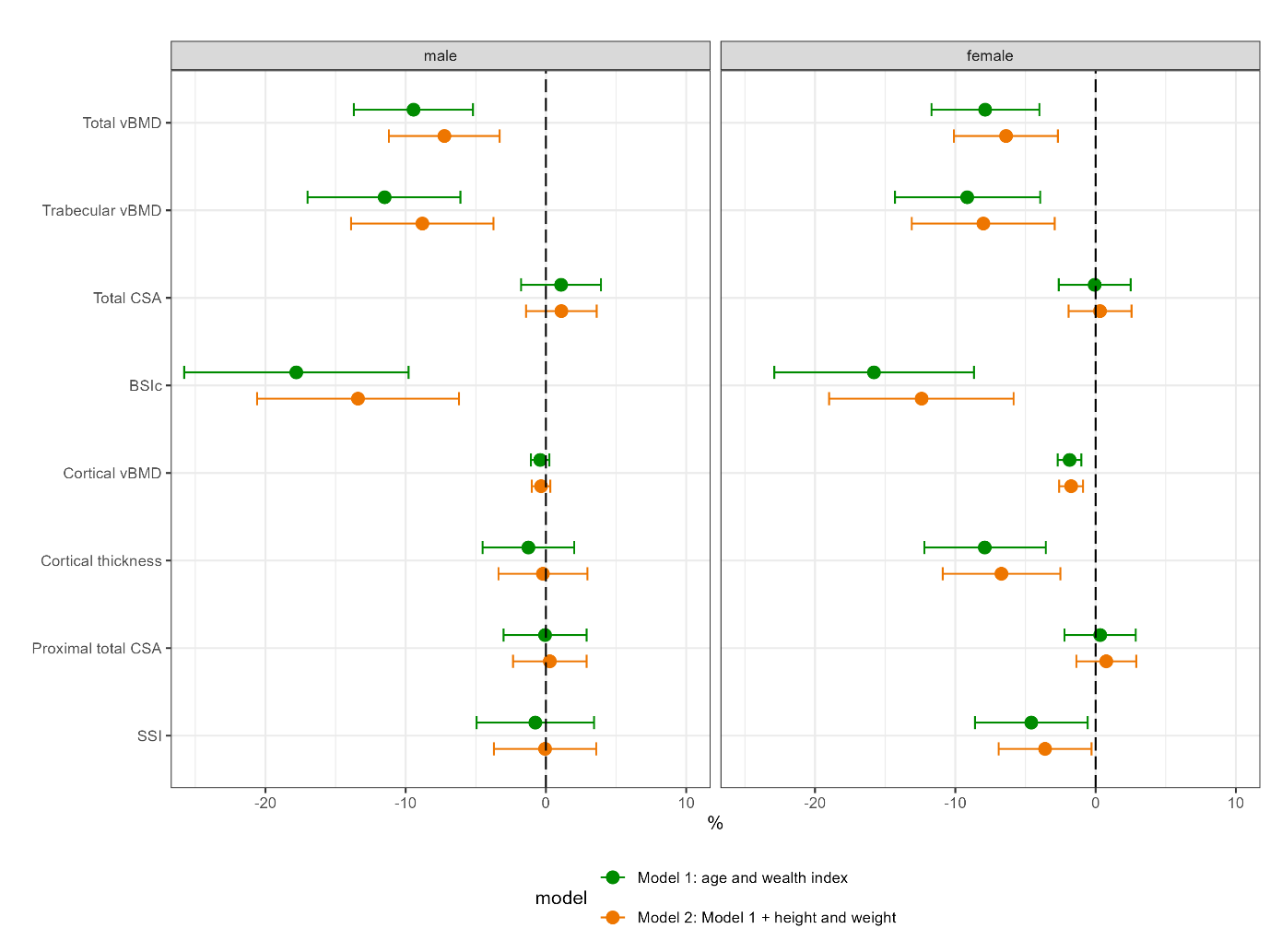


**Figure S5.** Associations between HIV-specific factors and tibial pQCT parameters in Zimbabwean men and women living with HIV, expressed as symmetric percentage differences. Panel A) Symmetric percentage difference in pQCT parameters per 1-year increase in time since HIV diagnosis, adjusted for age and wealth index (Model 1) and additionally for years of ART use (Model 2). Panel B) Symmetric percentage differences in pQCT parameters comparing people living with HIV by TDF use (TDF versus non-TDF regimen) and by viral load suppression status (suppressed, <50 copies/mL, versus unsuppressed), each adjusted for age and wealth index. vBMD = volumetric bone mineral density; CSA = cross-sectional area; BSIc = bone strength index compression; pSSI = polar stress-strain index.


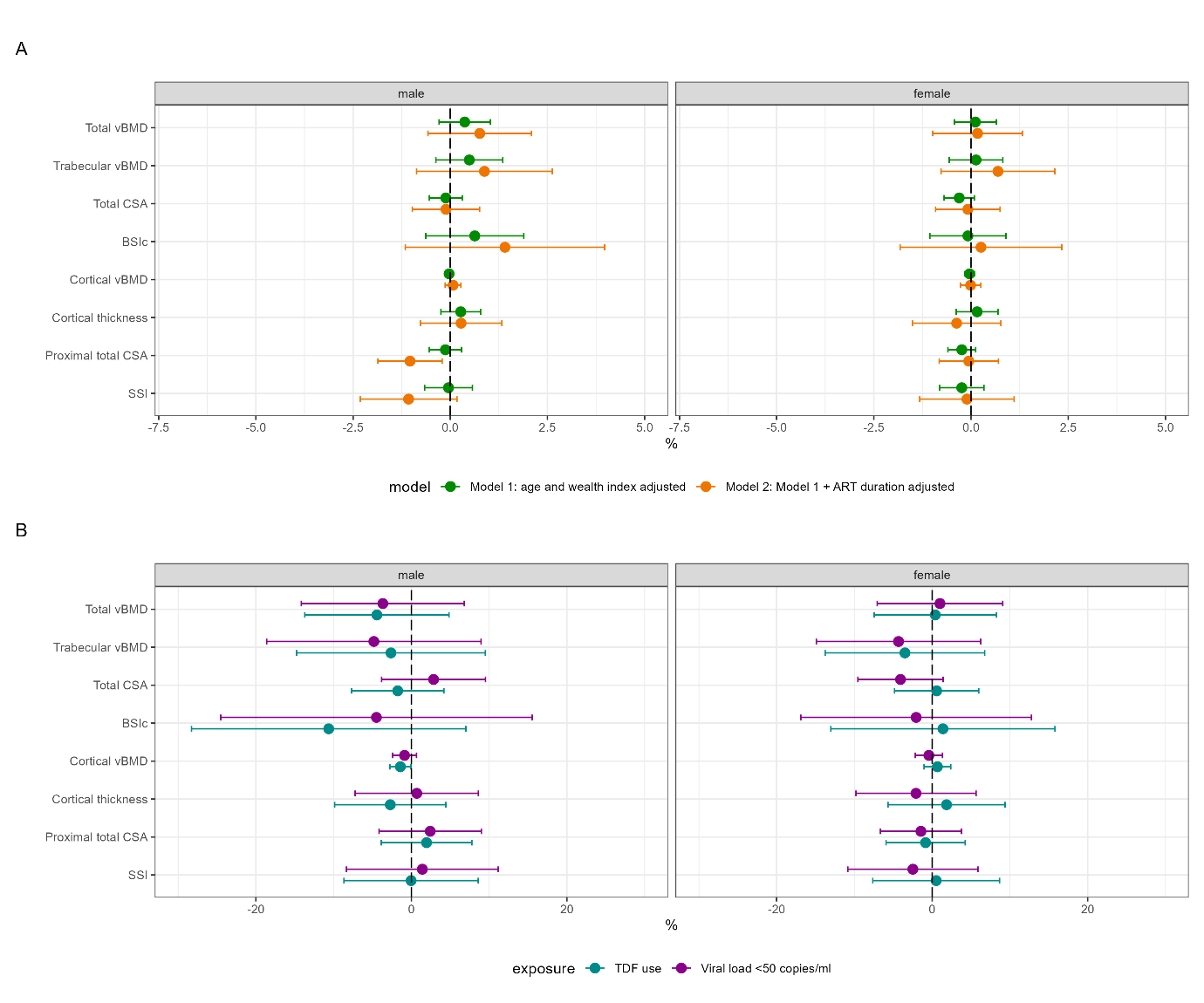


**Figure S6.** Differences in tibial pQCT parameters by major osteoporotic fracture (MOF) status in Zimbabwean older adults. Data expressed as mean(95% CI) sympercent difference between those with a history of MOF and those without. Model 1, unadjusted; Model 2 adjusted for age, sex, and wealth index. PLWH = people living with HIV vBMD = volumetric bone mineral density, CSA = cross-sectional area, BSIc = bone strength index of compression, SSI = stress-strain index.


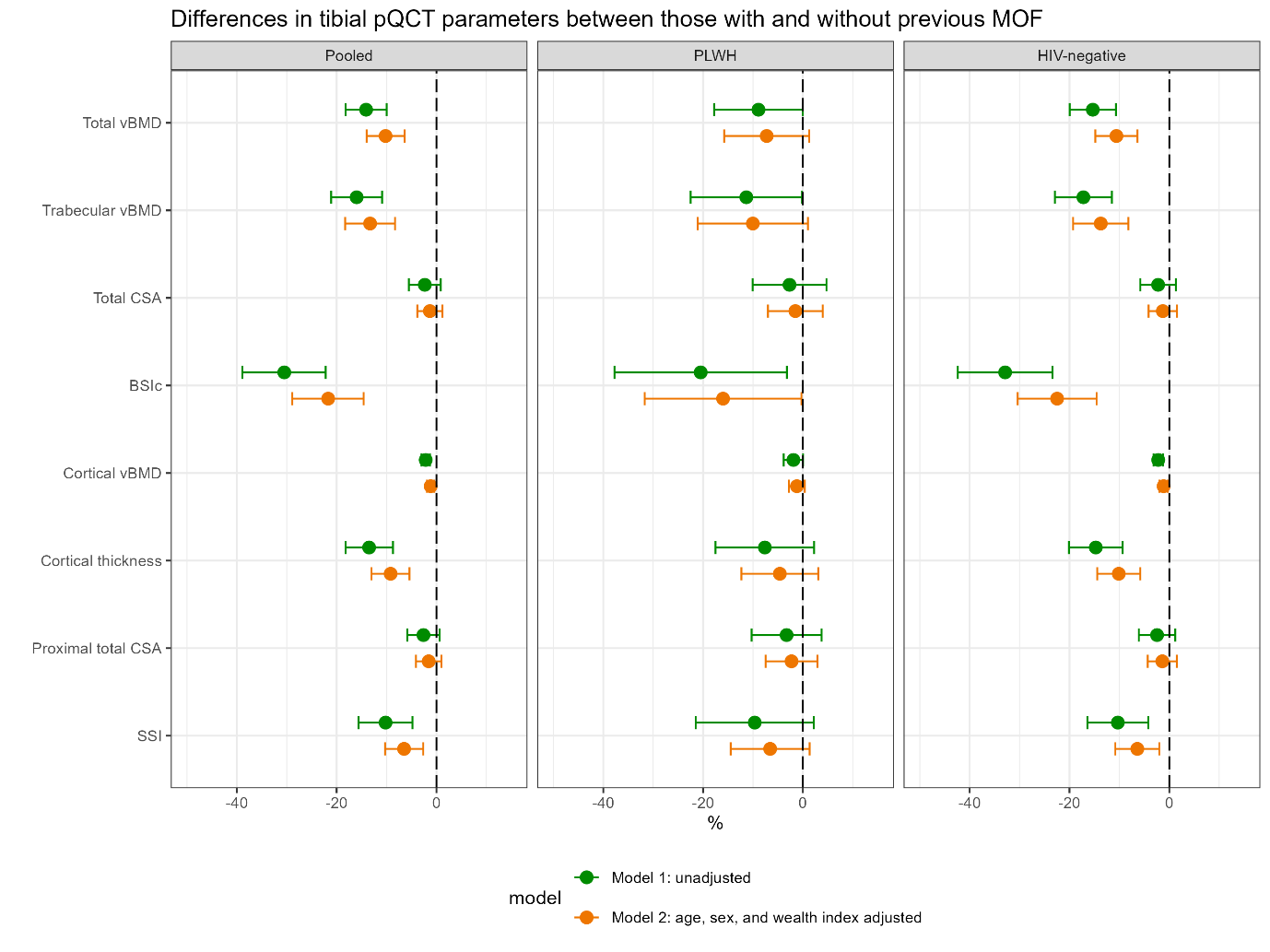


**Figure S7.** Differences in radial and tibial pQCT parameters by major osteoporotic fracture (MOF) status in Zimbabwean PLWH. Model 1, adjusted for age, sex, and wealth index; Model 2, Model 1 + time since HIV diagnosis; Model 3, Model 2 + years on ART. Data expressed as mean(95% CI) sympercent difference between those with a history of MOF and those without. PLWH = people living with HIV vBMD = volumetric bone mineral density, CSA = cross-sectional area, BSIc = bone strength index of compression, SSI = stress-strain index.


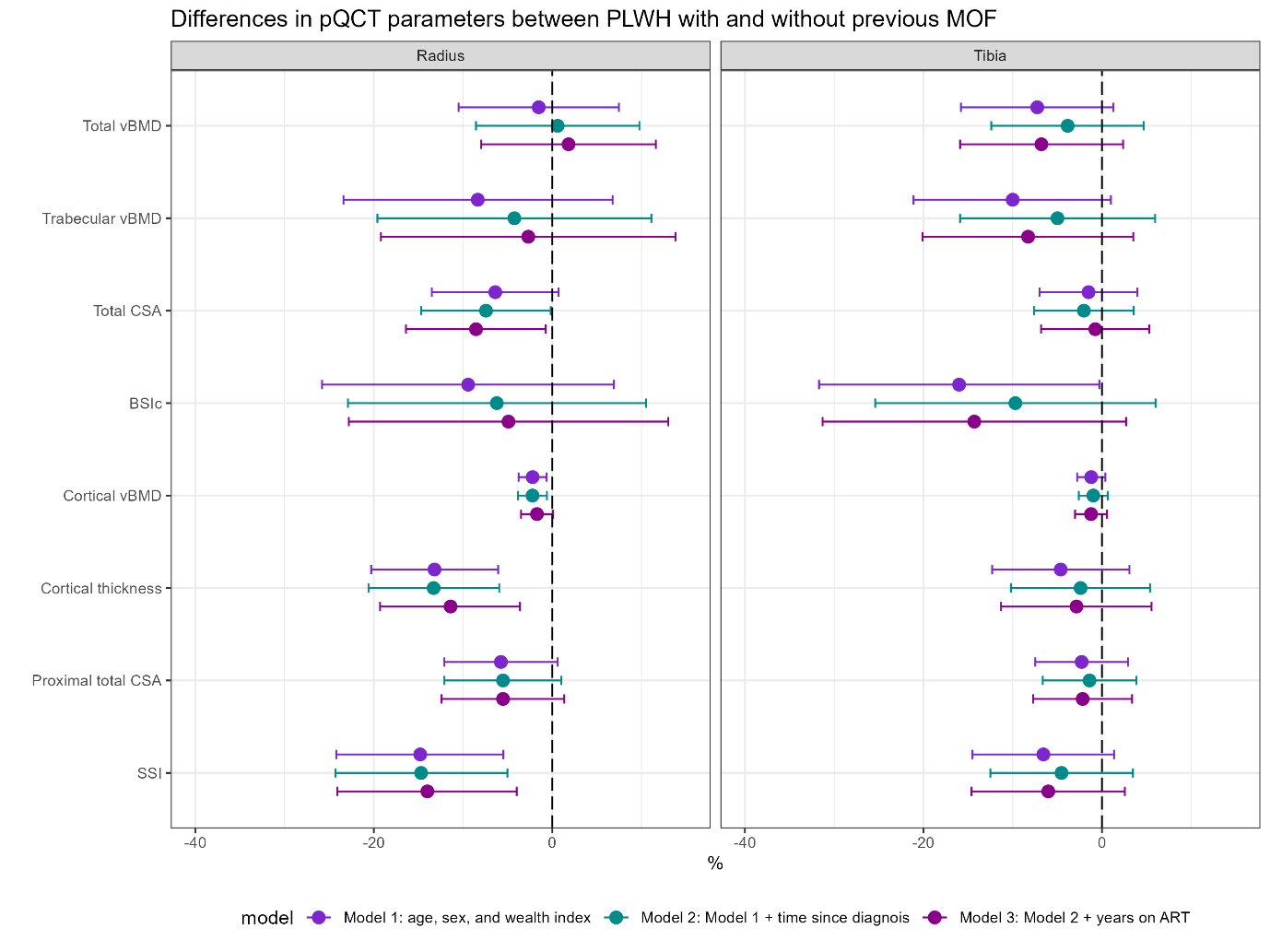

Supplement: MS_ASBMR-25090748_Supplementary_materials_2026_05_06_zjag082 [file ms_asbmr-25090748_supplementary_materials_2026_05_06_zjag082.docx]
