# Supplementary material for: Sex-specific bone architectural deficits among older adults living with HIV: a cross-sectional study from Zimbabwe
Source: J Bone Miner Res. 2026 May 12;41(8):848–58. doi: 10.1093/jbmr/zjag082 (PMC13421066; doi:10.1093/jbmr/zjag082)
Supplement: STROBE_Strengthening_the_Reporting_of_Observational_Studies_in_Epidemiology [file strobe_strengthening_the_reporting_of_observational_studies_in_epidemiology.pdf]

Journal of Bone and Mineral Research

JBMR®

2001 K Street, NW,
3rd Floor
NorthWashington,
DC 20006, USA

P: 202-367-2336
E: jbmr@asbmr.org

Corresponding Author Name: Mícheál Ó Breasail

Manuscript Title: Sex-specific bone architectural deficits among older adults living with H

Strengthening the Reporting of Observational Studies in Epidemiology

When reporting results of studies involving humans, the *JBMR*® adheres to the STROBE statement, and authors must provide the information required by the STROBE checklist as adapted by JBMR. Since statistical analyses vary according to study design and prohibit the establishment of standard rules, the following guidelines are provided to assist authors in performing the most appropriate statistical analyses and reporting those results in accord with *JBMR*® standards. The checklist aids authors in providing detailed information within their submission that meet a standard to achieve reproducibility and transparency of research, and assists reviewers in their effort determine whether the necessary information is present. If you have any questions, please contact us at jbmr@asbmr.org

JBMR® has adapted the STROBE Guidelines. To fulfill the guideline requirements for JBMR®, please select the statement that describes your study and fill out only the required STROBE checklist questions for the study.

- ☐ STROBE CHECKLIST A- I am reporting the results of a cohort study.
- ☐ STROBE CHECKLIST B- I am reporting the results of a case-control study.
- ☑ STROBE CHECKLIST C- I am reporting the results of a cross-sectional study.

STROBE CHECKLIST A

Cohort Study

Recommendation- Cohort Study	Page
Title/Abstract/Introduction- Indicate the study's design with a commonly used term in the title or the abstract. State specific objectives, including any prespecified hypotheses in introduction.	
Methods- Present key elements of study design early in the paper. Describe the setting, locations, and relevant dates, including periods of recruitment, exposure, follow-up, and data collection.<br>❖ Give the eligibility criteria, and the sources and methods of selection of participants. Describe methods of follow-up. For matched studies, give matching criteria and number of exposed and unexposed	
Clearly define all outcomes, exposures, predictors, potential confounders, and effect modifiers. Give diagnostic criteria, if applicable.<br>❖ For each variable of interest, give sources of data and details of methods of assessment (measurement). Describe comparability of assessment methods if there is more than one group.<br>❖ Describe any efforts to address potential sources of bias<br>❖ Explain how the study size was arrived at	
Explain how quantitative variables were handled in the analyses. If applicable, describe which groupings were chosen and why<br>❖ Describe all statistical methods, including those used to control for confounding. Describe any methods used to examine subgroups and interactions.<br>❖ Explain how missing data were addressed<br>❖ If applicable, explain how loss to follow-up was addressed<br>❖ Describe any sensitivity analyses	
Results- Report numbers of individuals at each stage of study—numbers potentially eligible, examined for eligibility, confirmed eligible, included in the study, completing follow-up, and analyzed<br>❖ Give reasons for non-participation at each stage<br>❖ Indicate number of participants with missing data for each variable of interest<br>❖ Summarize follow-up time (eg, average and total amount)	
Report numbers of outcome events or summary measures over time	
Give unadjusted estimates and, if applicable, confounder-adjusted estimates and their precision (eg, 95% confidence interval). Make clear which confounders were adjusted for and why they were included.<br>❖ Report category boundaries when continuous variables were categorized<br>❖ If relevant, consider translating estimates of relative risk into absolute risk for a meaningful time period<br>❖ Report other analyses done—e.g. analyses of subgroups and interactions, and	

Recommendation- Cohort Study	Page
sensitivity analyses	
Discussion- Discuss limitations of the study, taking into account sources of potential bias or imprecision. Discuss both direction and magnitude of any potential bias. Discuss the generalisability (external validity) of the study results	

STROBE CHECKLIST B

Journal of Bone and Mineral Research
JBMR

Case-control study

Recommendation- Case-control study	Page
Title/Abstract/Introduction- Indicate the study's design with a commonly used term in the title or the abstract. State specific objectives, including any prespecified hypotheses in introduction.	
Methods- Present key elements of study design early in the paper. Describe the setting, locations, and relevant dates, including periods of recruitment, exposure, follow-up, and data collection.<br>❖ Give the eligibility criteria, and the sources and methods of case ascertainment and control selection. Give the rationale for the choice of cases and controls. For matched studies, give matching criteria and the number of controls per case.	
Clearly define all outcomes, exposures, predictors, potential confounders, and effect modifiers. Give diagnostic criteria, if applicable.<br>❖ For each variable of interest, give sources of data and details of methods of assessment (measurement). Describe comparability of assessment methods if there is more than one group<br>❖ Describe any efforts to address potential sources of bias<br>❖ Explain how the study size was arrived at	
Explain how quantitative variables were handled in the analyses. If applicable, describe which groupings were chosen and why<br>❖ Describe all statistical methods, including those used to control for confounding. Describe any methods used to examine subgroups and interactions.<br>❖ Explain how missing data were addressed<br>❖ If applicable, explain how matching of cases and controls was addressed<br>❖ Describe any sensitivity analyses	
Results- Report numbers of individuals at each stage of study—numbers potentially eligible, examined for eligibility, confirmed eligible, included in the study, completing follow-up, and analyzed<br>❖ Give reasons for non-participation at each stage<br>❖ Indicate number of participants with missing data for each variable of interest	
Report numbers in each exposure category, or summary measures of exposure	
Give unadjusted estimates and, if applicable, confounder-adjusted estimates and their precision (eg, 95% confidence interval). Make clear which confounders were adjusted for and why they were included.<br>❖ Report category boundaries when continuous variables were categorized<br>❖ If relevant, consider translating estimates of relative risk into absolute risk for a meaningful time period<br>❖ Report other analyses done—e.g. analyses of subgroups and interactions, and sensitivity analyses	

Recommendation- Case-control study	Page
Discussion- Discuss limitations of the study, taking into account sources of potential bias or imprecision. Discuss both direction and magnitude of any potential bias. Discuss the generalisability (external validity) of the study results	

STROBE CHECKLIST C

Cross-sectional study

Recommendation- Cross-sectional study	Page
Title/Abstract/Introduction- Indicate the study's design with a commonly used term in the title or the abstract. State specific objectives, including any prespecified hypotheses in introduction.	2-4
Methods- Present key elements of study design early in the paper. Describe the setting, locations, and relevant dates, including periods of recruitment, exposure, follow-up, and data collection.<br>❖ Give the eligibility criteria, and the sources and methods of selection of participants	4
Clearly define all outcomes, exposures, predictors, potential confounders, and effect modifiers. Give diagnostic criteria, if applicable.<br>❖ For each variable of interest, give sources of data and details of methods of assessment (measurement). Describe comparability of assessment methods if there is more than one group<br>❖ Describe any efforts to address potential sources of bias<br>❖ Explain how the study size was arrived at	4-8
Explain how quantitative variables were handled in the analyses. If applicable, describe which groupings were chosen and why<br>❖ Describe all statistical methods, including those used to control for confounding. Describe any methods used to examine subgroups and interactions.<br>❖ Explain how missing data were addressed<br>❖ If applicable, describe analytical methods taking account of sampling strategy<br>❖ Describe any sensitivity analyses	7-8
Results- Report numbers of individuals at each stage of study—eg numbers potentially eligible, examined for eligibility, confirmed eligible, included in the study, completing follow-up, and analyzed<br>❖ Give reasons for non-participation at each stage<br>❖ Indicate number of participants with missing data for each variable of interest	8-9
Report numbers of outcome events or summary measures	8-9
Give unadjusted estimates and, if applicable, confounder-adjusted estimates and their precision (eg, 95% confidence interval). Make clear which confounders were adjusted for and why they were included.<br>❖ Report category boundaries when continuous variables were categorized<br>❖ If relevant, consider translating estimates of relative risk into absolute risk for a meaningful time period<br>❖ Report other analyses done—e.g. analyses of subgroups and interactions, and sensitivity analyses	9-11

Recommendation- Cross-sectional study	Page
Discussion- Discuss limitations of the study, taking into account sources of potential bias or imprecision. Discuss both direction and magnitude of any potential bias. Discuss the generalisability (external validity) of the study results	11-14
